# Screening of Endophytic Bacteria of *Leucojum aestivum* ‘Gravety Giant’ as a Potential Source of Alkaloids and as Antagonist to Some Plant Fungal Pathogens

**DOI:** 10.3390/microorganisms10102089

**Published:** 2022-10-21

**Authors:** Yuka Munakata, Rosella Spina, Sophie Slezack-Deschaumes, Julie Genestier, Alain Hehn, Dominique Laurain-Mattar

**Affiliations:** 1 Université de Lorraine—INRAE, LAE, F-54000 Nancy, France; 2 Université de Lorraine—CNRS, L2CM, F-54000 Nancy, France

**Keywords:** *Leucojum aestivum*, ‘Gravety giant’, endophytic bacteria, wheat fusarium, tryptophol, LC-MS

## Abstract

*Leucojum aestivum* is a medicinal plant belonging to the Amaryllidaceae family well known as a producer of alkaloids such as galanthamine and lycorine. However, the endophytic microbes that colonize different plant tissues without causing any damage have not been reported in this plant. Here, we explored the different endophytic bacterial communities isolated from different surface disinfected tissues of *L. aestivum* ‘Gravety giant’ and screened bacterial isolates producing alkaloids and their potential use as biocontrol agent against wheat pathogens. For that purpose, endophytic bacteria were isolated from bulbs, roots and shoots of *L. aestivum*. After taxonomical characterization, these microorganisms were screened for their ability to produce alkaloids using high-performance thin-layer chromatography (HPTLC) and untargeted liquid chromatography-Mass Spectrometry/Mass Spectrometry (LC-MS/MS) strategies. We isolated 138 bacteria belonging to four phyla and 42 genera, mainly from roots and shoots. The most abundant genera were Rahnella in shoot, Patulibacter in bulb and Bacillus in roots. Among the different bacterial isolates, the methanolic extracts of *Luteibacter rhizovicinus* (LaBFB3301) and *Commamonas denitrificans* (LaBFS2103) slightly delayed the growth of *F. graminearum* colonies in in vitro dual tests against *F. graminearum* and *M. nivale* strains with 15.5% and 19.9% inhibition rates, respectively. These isolates are able to produce an indolic alkaloid tryptophol (C_10_H_11_NO, [M + H]^+^ 162.0913). These endophytic bacteria might be investigated to characterize the plant protection effect and the plant growth promotion effect.

## 1. Introduction

Endophytic microbes are found in all plant species studied so far [1]. Although endophytes involve archaea, protists and viruses, most of them are represented by bacteria and fungi. Even though fungal endophytes have been more intensively studied than bacterial endophytes, such bacterial endophytes are of interest in terms of plant growth promotion effects and the robustness of the plant immune system against fungal/oomycete plant pathogens [2]. These bacteria are also able to produce bioactive specialized metabolites with various biological activities, and endophytes may modulate plant metabolite synthesis. However, the metabolic interactions between plants and endophytes remain largely unknown [3]. A great number of endophytic bacteria have been reported, including Proteobacteria, Actinobacteria, Bacteroidetes and Firmicutes [4,5,6]. The population densities of endophytic bacteria are extremely variable considering plant species and organs [7]. Roots and below-ground tissues tend to yield the highest density of bacteria compared to above-ground tissues, while there are a small number of reports about endophytes in flowers, fruits and seeds [8,9].

The microbes isolated from inner plant tissues are attractive and might constitute original factories for producing new bioactive molecules [10,11,12]. Furthermore, some endophytes have also been reported to mimic the synthesis of plant compounds, such as paclitaxel and vinblastine [1,13,14]. It is known that these microorganisms are also able to produce different specialized metabolites such as non-ribosomal synthesized peptides, polyketides, alkaloids, sesquiterpenes, flavonoids, saponins, lactones and organic acids [15,16], which confer ecological benefits to their host plants. These compounds can possess pharmaceutical functions such as antifungal, antibacterial, antivirus, anticancer as well as anti-inflammatory properties. Bioactive molecules such as actinoallolides A (anti-trypanosomal activity) [17], ecomycins (antifungal activity) [18] and maytansine (cytotoxicity) [19] were discovered and isolated from bacterial endophytes. It was also demonstrated that a *Bacillus* strain isolated from an Icacinaceae plant was also able to produce camptothecin, a quinoline alkaloid, independently from the host plant [14,20]. Some specialized metabolites isolated from endophytic bacteria have shown agronomical interests such as phenolic compounds, volatile organic compounds that show phytotoxic activity [21], and antifungal and nematicidal properties [22,23]. These examples clearly establish that endophytic bacteria might be used as bioreactors or as genomic resources to create cell factories for producing phytochemicals independently of their host plants or to characterize new leads for further agrochemical and pharmaceutical applications.

Bulbous plants of the Amaryllidaceae family are known for the synthesis of isoquinoleic alkaloids, also known as Amaryllidaceae alkaloids, including lycorine and galantamine [24]. These alkaloids exhibit a wide range of biological activities, such as acetylcholinesterase inhibition for galantamine, which led to their use as drugs. Only a few studies on the molecular identification of endophytes isolated from Amaryllidaceae plants and the identification of compounds extracted from these endophytic microorganisms were reported. Hexacyclopeptides have been identified in the endophytic fungus *Fusarium salani* from *Narcissus tazetta* [25]. From *Leucojum aestivum*’s in vitro bulblets, five Amaryllidaceae alkaloids, four amino acids and fatty acids were identified in the endophytic bacteria belonging to the *Bacillus* genus [26]. In this study, we analyze endophytic bacteria from bulbs, roots and shoots of *L. aestivum* ‘Gravety giant’. Because it is reported that Amaryllidaceae alkaloids differently accumulate depending on plant tissues [27], we assumed that the different tissues host different endophytic communities in terms of taxonomy and metabolites. We screened bacterial isolates with Dragendorff’s reagent for the detection of alkaloids on HPTLC plates and performed fine LC-MS/MS analysis of methanolic extracts from bacterial pellets. The isolates were taxonomically identified using 16S RNA sequencing, and their potential activities towards some wheat fusarium wilt agents such as *Fusarium graminearum* and *Microdochium nivale* were investigated.

## 2. Materials and Methods

### 2.1. Plant Material and Culture Conditions

The soil used was a clay soil (FAO-WRB classification) sampled from the 0–20 cm layer at the Bouzule research farm in northeastern France (48°74′ N, 6°35′ E). The bulbs of *Leucojum aestivum* ‘Gravety giarance). The bulbs were sont’ were purchased from a French market (Pépinière Lepage ‘Val de Loire’, Les Ponts-de-Cé, Fwn in a container (76 cm × 38.5 cm × 23 cm, 5 kg of soil) placed indoors and watered with 2 L of water once a week at 20 °C with a 16/8 h light/dark cycle until bacterial isolation. After 90 days, 3 plants showing shoots about 10 cm long were harvested.

The plant materials were washed with running tap water and the different tissues were separated using blades into aerial parts (shoots), bulbs and roots. Shoot and root parts were washed again with tap water and then dried on paper towels. For bulbus parts, the outer tunic was peeled by blade and by hand, and the basal plate was removed by blade because of their difficulty for disinfection. The treated bulb was cut in half and individual scales were separated by hand. Each tissue material was weighed just after sampling. The fresh materials were then cut into certain sizes: shoot and bulbus materials were 1 to 1.5 cm in horizontal length and 2 to 3 cm in vertical length; root materials were around 3 cm in length. The trimmed materials were randomly distributed by around 1.1 g in a sterile 50 mL tube and stored at 4 °C. They were used for bacterial isolation within 48 h after being harvested.

### 2.2. Bacterial Isolation and Cultivation

The plant materials were processed as described by Munakata et al. [28], with a minor modification depending on the plant tissue. Briefly, for the surface sterilization, the shoot materials were treated in 70% ethanol for 1 min and in 2.6% bleach solution for 2.5 min; the bulb materials were treated in 70% ethanol for 1 min and in 2.6% bleach solution for 5 min; the root materials were treated in 70% ethanol for 1 min 3 times and in 2.6% bleach solution for 5 min. At last, all the materials were rinsed in sterile ultrapure water for 1 min, 3 times. All the treatments and the rinses were performed in 20 mL solution with agitation. The final rinse solution was used for testing the efficiency of the surface sterilization by spreading 100 µL on 10% Tryptic soy agar (10% TSA) media (Sigma-Aldrich, St. Quentin Fallavier, France) containing cycloheximide at 100 mg/L (10% TSAC). The treated plant materials were cut into smaller pieces using a sterile blade and then grinded by sterile tubes on sterile Petri dishes. They were then incubated in 10 mL of 0.85% (*w/v*) sodium chloride for 1 h with agitation at 60 rpm at 28 °C. Then, 100 µL of the incubated suspension was spread on three plates of 10% TSAC, with a dilution series of 1 time and 10 times. The plates were incubated at 28 °C for 20 days. The number of colonies was expressed as CFU/g of fresh plant material. Colonies were purified by repeated sub-culturing, and single colonies were picked for subsequent analysis. The pure isolates were also stored in 15% glycerol solution at −80 °C.

### 2.3. Molecular Identification

The bacteria were taxonomically identified by PCR amplification of the 16S rDNA regions, performed with a method described in Munakata et al. [28]. For some isolated bacteria, DNA was sequenced using the DNeasy^®^ UltraClean^®^ Microbial Kit (QIAGEN, Hilden, Germany) and used as a template for PCR amplification with the primer sets of 27F (5′–GAGAGTTTGATCCTGGCTCAG–3′) and 1492R (5′–CTACGGCTA CCTTGTTACGA–3′). The PCR 50 µL reaction mix contained 1 µL of PrimeSTAR^®^ GXL DNA Polymerase (Takara, St. Germain-en-Laye, France), 10 µL of 5×PrimeStar GXL buffer, 4 µL of dNTPs (2.5 mM for each), 1 µL of each primer at 10 µM and 1 µL of genomic DNA. PCR amplification was performed using the following program: 10 s 98 °C, followed by 30 cycles of 10 s 98 °C, 15 s 55 °C, 2 min 68 °C, with the final elongation at 68 °C for 2 min. PCR products were commercially sequenced (Eurofins, Nantes, France). DNA sequences were edited and screened against those in the GenBank database using BLAST (http://www.ncbi.nlm.nih.gov/ accessed on 20 September 2022). The sequences have been deposited in GenBank under accession numbers (OL307013 to OL307079, accessed on 31 October 2021), shown in Table S1.

### 2.4. Crude Extract Preparation of Bacterial Pellet

Crude extracts for alkaloid detection were prepared from bacterial pellets. A preculture of the bacteria was performed in 2 mL nutrient broth (NB) (Sigma) at 28 °C and 180 rpm for 24 h. Then, 100 µL of the precultures was added to 50 mL NB in 250 mL Erlenmeyer flasks. After 72 h of cultivation at 28 °C 180 rpm, the bacterial pellets were collected by centrifugation at 4000× *g* at room temperature for 40 min. The pellets were suspended in 1.5 mL methanol and treated with ultrasonication for 30 min, followed by maceration for 30 min at room temperature. The supernatant was then obtained by centrifugation at 12,800× *g* at room temperature for 10 min.

### 2.5. High-Performance Thin-Layer Chromatography (HPTLC) Analysis and Alkaloid Detection with Dragendorff’s Reagent

Briefly, 20 µL of the extracts and 5 µL of a standard mixture of lycorine and galantamine at 1 mg/mL each were sprayed with LINOMAT 5 (Camag^®^, Muttenz, Switzerland) onto HPTLC plates 60 F_254_ (Merck, Darmstadt, Germany), 20 × 10 cm. The plates were developed in the mobile phase consisting of 10 mL of ethyl acetate, 1.1 mL of acetic acid, 1.1 mL of formic acid and 2.5 mL of ultrapure water. After the development, the plates were air-dried. To screen the presence of alkaloids, the plate was sprayed with fresh Dragendorff’s reagent containing a mixture of solution A, solution B, acetic acid and water (1:1:4:20 *v/v*). Herein, solution A was 0.85 g basic bismuth nitrate in 10 mL acetic acid and 40 mL ultrapure water; solution B was 8 g potassium iodide in 30 mL ultrapure water. The colorized plates were observed successively just after spraying, after 30 min and after one day. The orange coloration of a spot indicated the potential detection of an alkaloid. The standards galanthamine and lycorine were purchased from Medchem Express, tryptophol from VWR and indole-3-acetic acid from Sigma-Aldrich (St. Quentin Fallavier, France).

### 2.6. Dual-Culture Assay to Test the Antagonistic Activity of Endophytic Bacteria against Two Cereal Pathogens

The antagonistic activities were analyzed using an in vitro dual-inoculation method. *F. graminearum* strain was kindly provided by Institut Français de la Brasserie et de la Malterie (IFBM), and *M. nivale* strain (MUCL 18523) was purchased from BCCL/MUCL. They were cultivated on potato dextrose agar (PDA) (Sigma) at 28 °C for *F. graminearum* and at 20 °C for *M. nivale* in darkness. Mycelial plugs of these fungi, 8 mm in diameter, were prepared from an active growing zone of the fresh cultures. One plug was inoculated at the center of a plate containing 25 mL of PDA. Then, 50 µL of a bacterial suspension obtained from a liquid culture of a bacterial isolate in 2 mL of NB at 28 °C for 24 h or 72 h was dropped on 4 spots that were 3 cm from the center of the plates. Three replicates were made for each bacterial isolate. The plates were incubated at 28 °C for *F. graminearum* and at 25 °C for *M. nivale* until the fungal colonies reached the edge of the plate. The diameter of the fungal colony was measured and averaged among replicates. Plates inoculated with fungi without bacterial cultures were used as controls. Antagonistic activities were expressed as the inhibitory rate (%), calculated as follows: ((d_control_ − d_assay_)/d_control_ × 100), where d is the diameter of the colony of the fungi.

### 2.7. Diffusion Assay of Cell-Free Culture Broth and Methanolic Extracts of the Selected Strains against F. graminearum mycelia

The crude extracts of bacterial isolates were prepared as described above. The 24-h-cultured bacterial broth in 2 mL NB was centrifuged at 15,000 rpm for 10 min and filtrated with Acrodisc^®^ Syringe Filter 0.2 µm Supor^®^ Membrane. For the diffusion assay, φ5.3 mm paper discs (Whatman, Merck, Darmstadt, Germany) were autoclaved and dried at 70 °C. Then, 25 µL of each sample was applied to one paper disc and the discs were dried under a laminar flow hood. Four discs per 20 mL PDA plate were placed diagonally 1 cm from the center. Fungal plugs were prepared and placed at the center as described above. The plates were incubated at 28 °C for *F. graminearum* and 20 °C for *M. nivale*. The diameters of the fungal colonies were measured every day from day 3. The growth inhibition rate was calculated as in the previous section.

### 2.8. LC-MS Equipment and Analysis

Untargeted LC-MS analysis was performed with UHPLC-Q-Exactive-Orbitrap (Thermo Fisher Scientific, Waltham, MA, USA). The column was Kinetex XB-C18 (150 × 2.1 mm × 2.6 µm) (Phenomenex, Le Pecq, France). The mobile phase consisted of 0.1% formic acid in water (A) and 0.1% formic acid in methanol (B) with 200 µL/min. Gradient elution was performed with the following program: 0–1 min, 10–10% B; 1–15 min, 10–60% B; 15–30 min, 60–95% B; 30–35 min, 95–95% B. MS data were obtained from an Orbitrap IDX mass spectrometer (Thermo Fisher Scientific, Waltham, MA, USA) using positive ionization mode. The ionization was achieved with the heated electrospray ionization (HESI) source and the scan range was 120 to 1200 *m/z*. The spectrometer worked with the data-dependent scan. MS2 fragments were obtained with collision-induced dissociation (HCD) at 30% energy. The resolution was 60,000 for MS1 and 30,000 for MS2.

The results were analyzed by Compound Discoverer 3.1 (Thermo Fisher Scientific, Waltham, MA, USA). The peaks were extracted with the threshold at minimum intensity = 50,000 *m/z* and then aligned and identified for metabolites, comparing with public databases such as mzCloud (https://www.mzcloud.org/ accessed on 20 September 2022). The peaks having a full match in mzCloud were used for further analysis, and categorized as ‘enriched’ in test samples when they satisfied log2 fold change to medium control >1 and *p* < 0.05.

To identify tryptophol and confirm the absence of lycorine, galanthamine and IAA, the bacterial methanolic extracts were analyzed by the LC-MS/MS system using positive ionization mode. It was realized by ESI high-resolution mass spectrometry (HRMS) with micrOTOF_Q_^TM^ (Bruker Daltonics, Bruker, Bremen, Germany) apparatus. The column was Hypersil gold (100 mm × 3 µm) (Thermo Scientific, Bellefonte, PA, USA) and the flow rate was 200 µL/min using the following program: 5% solvent B for 5 min, a gradient from 5% solvent B at 5 min to 99% of solvent B at 40 min, followed by 99% solvent B for 5 min. Solvent A was composed of 1% formic acid in water with LC/MS grade, and solvent B was composed of pure acetonitrile. The calibration was performed with a peak of sodium formate in each analysis. The spectrometer worked with the auto MS/MS mode. Each detected mass peak was isolated and fragmented with collision energy at 20 eV. The results were analyzed by Bruker Compass^®^ software.

### 2.9. Data Analysis

R studio with R version 3.6.3. was used for statistical tests and analysis. The Bray–Curtis distance was calculated with the R package ‘vegan’. This package was also used for performing PERMANOVA (permutations = 10,000) with adonis. Principal coordinate analysis (PCoA) and scatter plots were illustrated using the ggplot2 package. The Tukey–Kramer test was performed with the R package ‘multcomp’.

## 3. Results

### 3.1. Bacterial Collection Isolated from L. aestivum ‘Gravety Giant’

**Figure 1:**
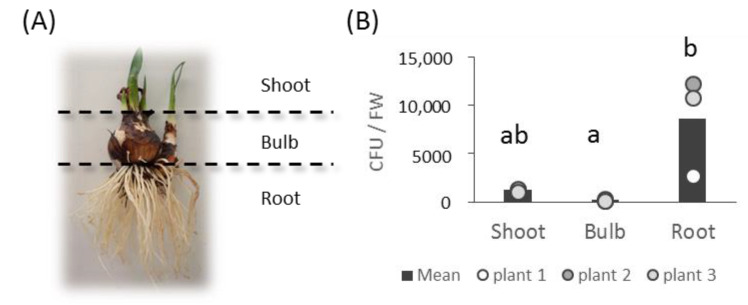
A used plant (plant 2) (**A**) and the number of colonies on TSA medium from 1 g of each fresh material after 20 days (**B**). CFU was tested with the Tukey–Kramer test, and the same letters (a, b) have no significant difference with *p* < 0.05.

Bacteria were isolated from three different organs of *L. aestivum* ‘Gravety giant’ plants at the vegetative stage, showing leaves about 7 cm long (Figure 1, Figure S1). The average density of culturable bacterial endophytes varied depending on the plant organs and was higher in roots (8569 colony-forming units per 1 g of fresh material weight (CFU/FW) compared to shoots (1210 CFU/FW) and bulbs (169 CFU/FW) (Figure 1).

### 3.2. Analysis of the Composition of Cultivable Endophytic Bacteria from L. aestivum ‘Gravety Giant’

**Figure 2:**
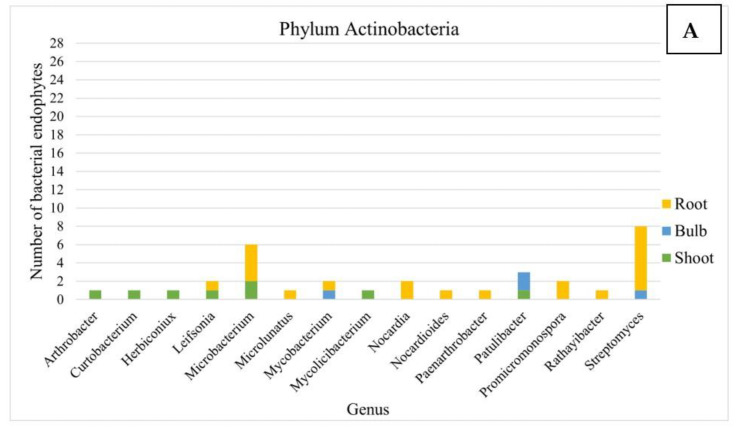

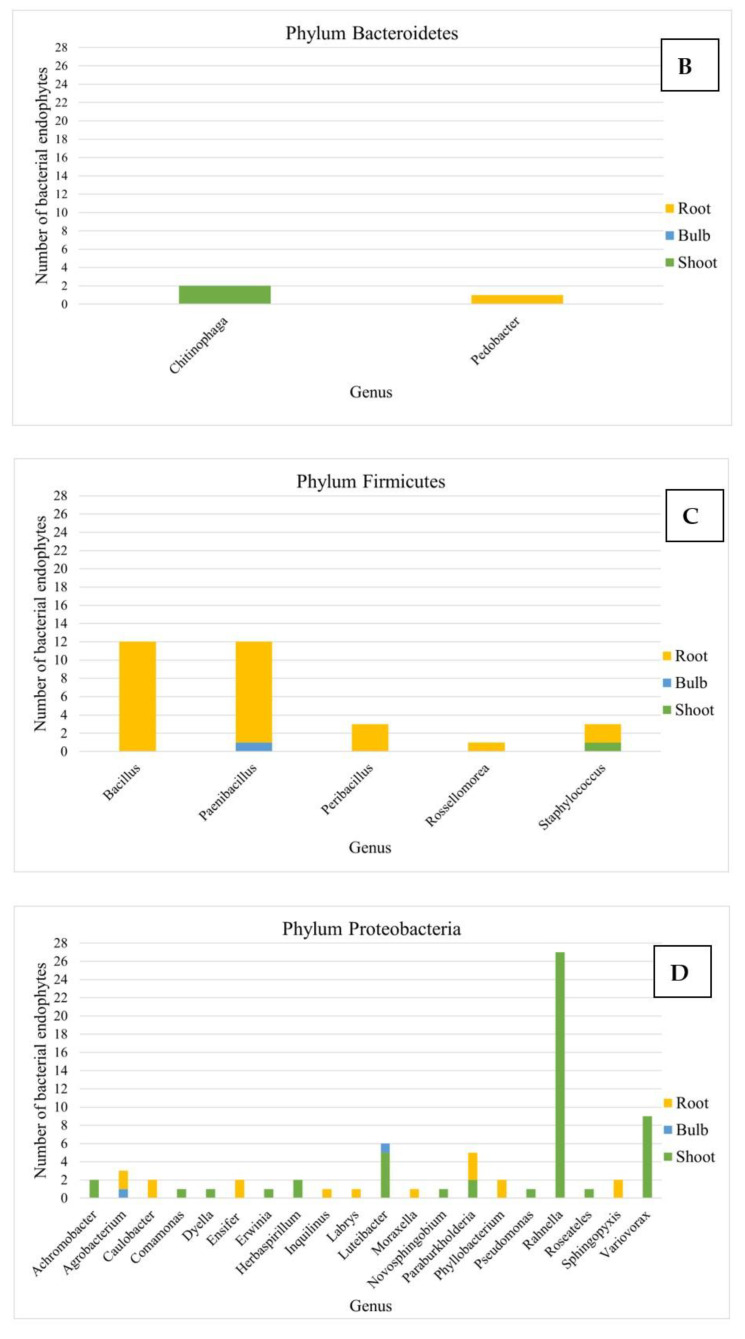
Taxonomical composition of the bacterial collection isolated from *L. aestivum* ‘Gravety giant’. (**A**) Phylum Actinobacteria, (**B**) Phylum Bacteroidetes, (**C**) Phylum Firmicutes, (**D**) Phylum Proteobacteria.

A total of 138 bacteria were successfully isolated and purified (Figure 2, Table S1). This bacterial collection consisted of 67 strains isolated from roots, 64 from the shoot material and only 7 from bulbs.

**Figure 3:**
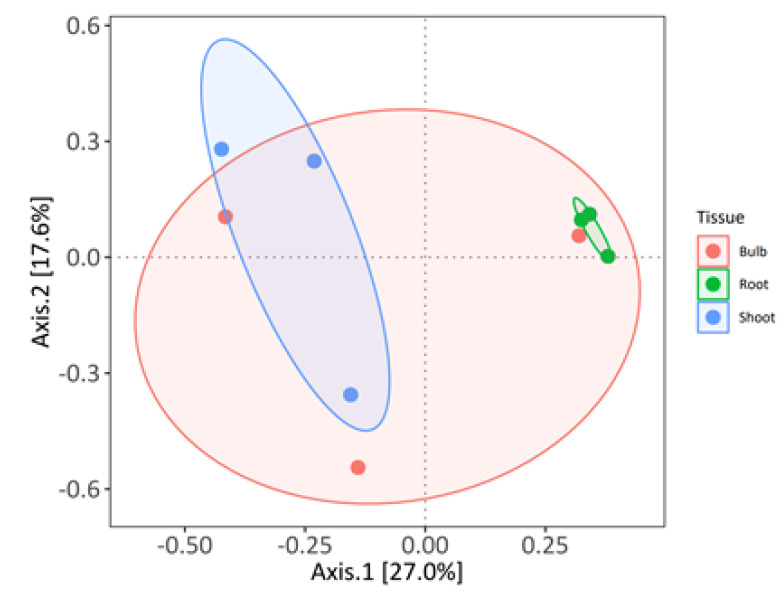
Principal coordinate analysis (PCoA) with Bray–Curtis distance of the *L. aestivum* ‘Gravety giant’ bacterial collections at the genus level.

The strains were taxonomically characterized after partial 16S rDNA sequencing. A total of 42 genera belonging to four phyla were identified. To assess the relative abundance of the compositions of the isolates from the three tissues at the genus level, we conducted a principal coordinate analysis (PCoA) using Bray–Curtis distance (Figure 3).

The first two axes explained 34.7% of the variation. The analysis showed that the bacterial communities from roots and shoots were well separated along the first axis, whereas the bacterial community from bulbs was not discriminated from the two other communities. This observation was validated by permutation ANOVA (permutations = 10,000), suggesting that the culturable bacterial communities from *L. aestivum* ‘Gravety giant’ are structured by plant organs (*p*-value = 0.0351).

**Figure 4:**
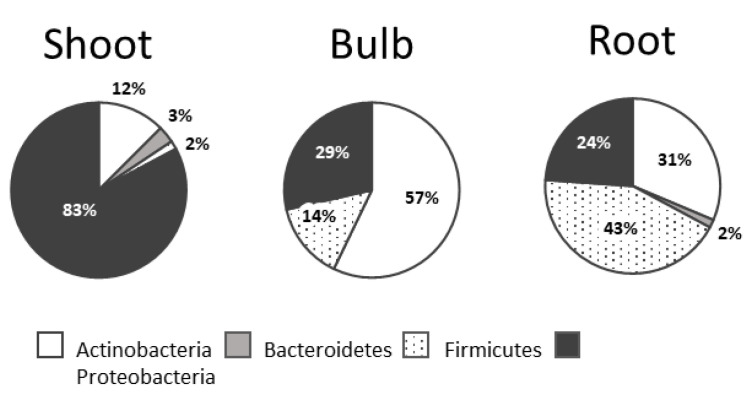
Relative abundance of each phylum in the bacterial collection of the different plant tissues.

Proteobacteria were dominant in shoots (83%), whereas Firmicutes were mainly detected in roots (43%) (Figure 4).

**Figure 5:**
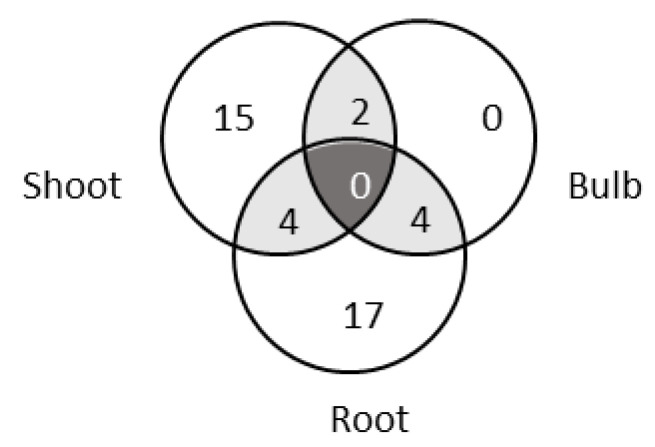
Venn diagram of shared and unique bacterial genera in *L. aestivum* ‘Gravity giant’.

At the genus level, 21 genera, 25 genera and 6 genera were found in the shoots, the roots and the bulbs, respectively. However, no genus was common among all three plant tissues (Figure 5).

The isolates from the bulbs were affiliated to six genera, which were shared with either shoot (two genera) or root (four genera) parts, and no genera were unique in bulbs. In shoots, the most abundant genera were *Rahnella*, *Variovorax* and *Luteibacter*, while in roots, *Bacillus*, *Paenibacillus* and *Steptomyces* were the dominant genera (Figure 2).

### 3.3. Alkaloid Screening Using High-Performance Thin-Layer Chromatography (HPTLC) Analysis

Endophytic bacteria extracts were investigated for their phytochemical characteristics. For this purpose, the methanolic extracts obtained from bacterial pellets were screened for their potential to produce alkaloid using Dragendorff’s reagent after the development of HPTLC plates (Figure S2). Over time after the Dragendorff spraying, some orange signals were observed. Among 69 tested strains, around 14 isolates showed some clear coloring reaction for Dragendorff’s reagent (Table 1), but none of these molecules had the same Rf value as the standard compounds of lycorine and galantamine, which are known Amaryllidaceae alkaloids produced by *L. aestivum*.

### 3.4. Antifungal Activities of the Isolates

**Figure 6:**
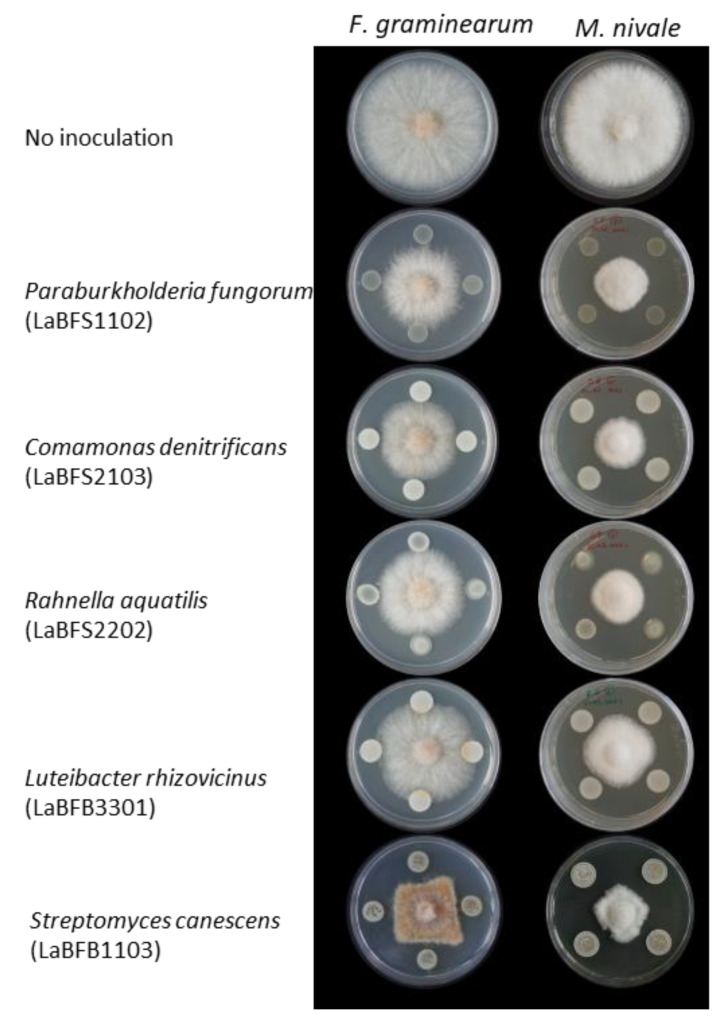
Dual-culture test of representative strains.

To evaluate the capabilities of bacterial isolates which reacted with Dragendorff’s reagent to inhibit *F. graminearum* and *M. nivale* mycelial growth, in vitro dual-culture tests were performed (Table 1, Figure 6). For strains affiliate to the same genus, such as *Rahnella*, we chose to test only a few strains from the same taxon (Table S1). Moreover, the isolates which required more than three days for sufficient growth in the liquid culture were removed from the dual-culture test.

**Figure 7:**
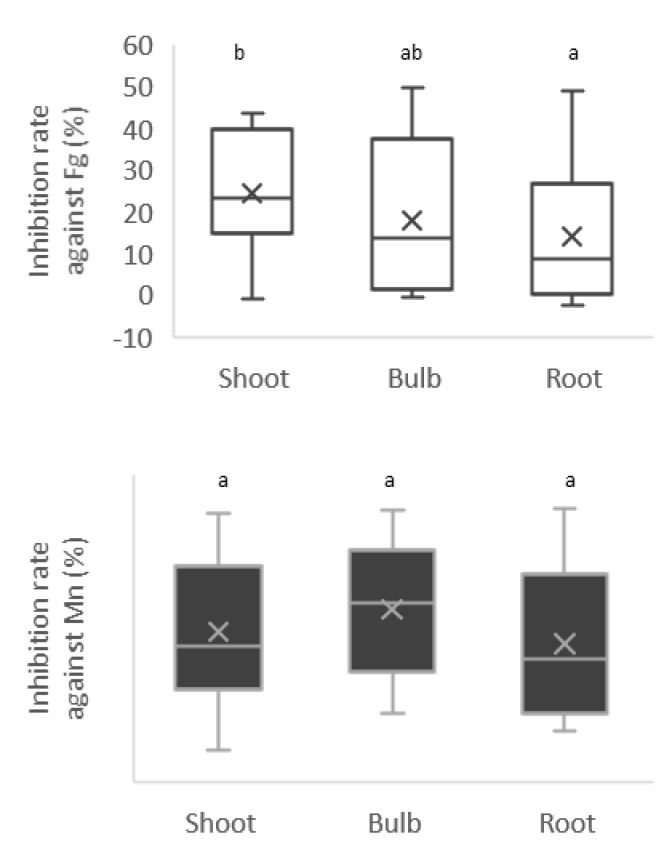
Antifungal activities against *F. graminearum* differed between the groups of shoot and root bacterial collections, but there was no difference in the antifungal activities for *M. nivale*. Different letters (a, b) show significant difference with *p* < 0.05 by the Tukey–Kramer method.

The antagonistic activities of the isolates against *F. graminearum* varied from 0% to 49.6% and differed between the bacterial group from shoot and root (Tukey–Kramer method, *p* = 0.00817) (Figure 7).

**Figure 8:**
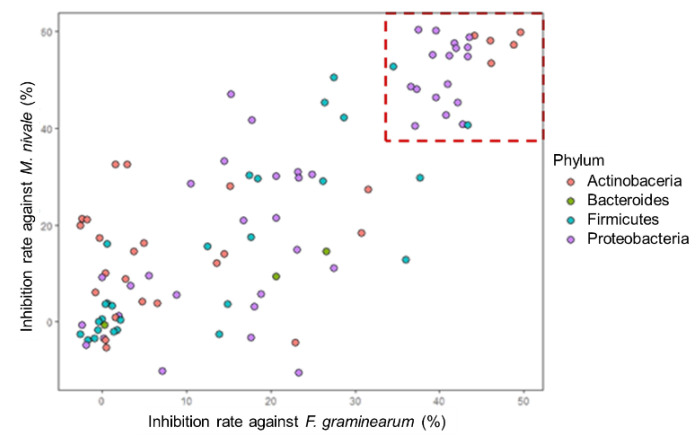
Correlations between antifungal activities against *F. graminearum* and *M. nivale*, colored by taxonomical affiliation at the phylum level.

On the other hand, those against *M. nivale* had a variation from 10.6% to 60.4%, but no significant differences were related to the source of plant tissues. Among them, 24 isolates, indicated in a red square in Figure 8, showed more than 30% inhibition against *F. graminearum* and more than 40% against *M. nivale*. This group contained 16 Proteobacteria strains (5 *Paraburkholderia*, 1 *Comamonas*, 8 *Rahnella*, 1 *Ensifer*, 1 *Luteibacter*, 1 *Erwinia*), 5 Actinobacteria strains (*Streptomyces*) and 2 Firmicutes strains (*Bacillus*).

**Figure 9:**
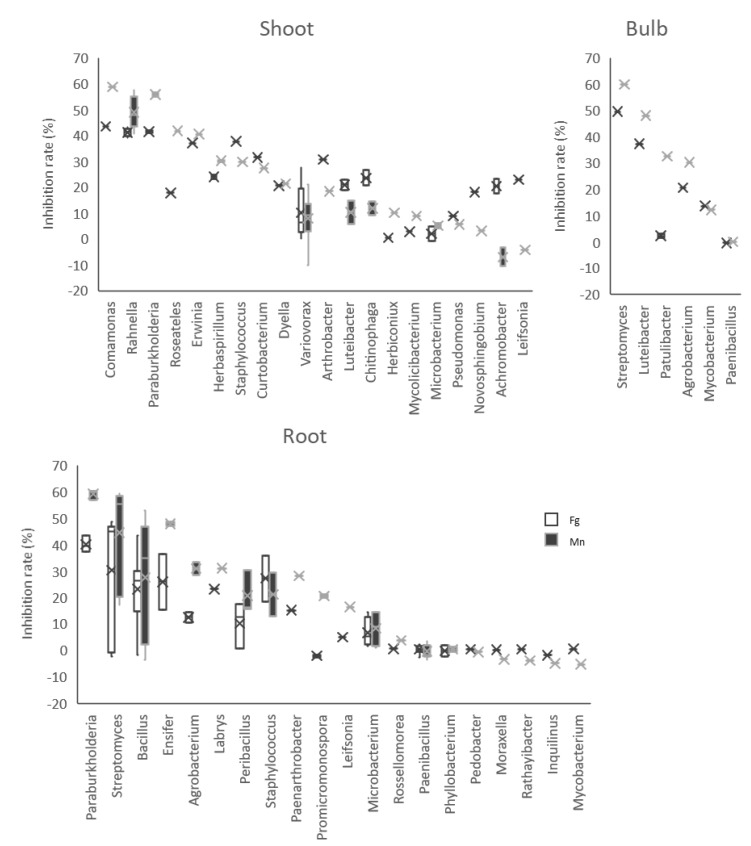
Growth inhibition rate against *F. graminearum* (Fg) and *M. nivale* (Mn) by genus of bacterial isolates in three different tissues: shoot, bulb and root.

The bacterial genera with the highest antifungal activities were different depending on the origin of plant tissues. In the shoot group, three Proteobacteria genera, namely *Comamonas*, *Rahnella* and *Paraburkholderia*, had a higher inhibition rate than the others, while it was *Streptomyces* in the bulb. *Paraburkholderia*, *Streptomyces* and *Bacillus* had better antifungal activities in the root group (Figure 9). Interestingly, *Paraburkholderia*, which appeared in the shoot and root parts, showed higher antagonisms with less variation among the isolates.

### 3.5. Antagonism Activities of Cell-Free Supernatant and Methanolic Extracts against F. graminearum

**Figure 10:**
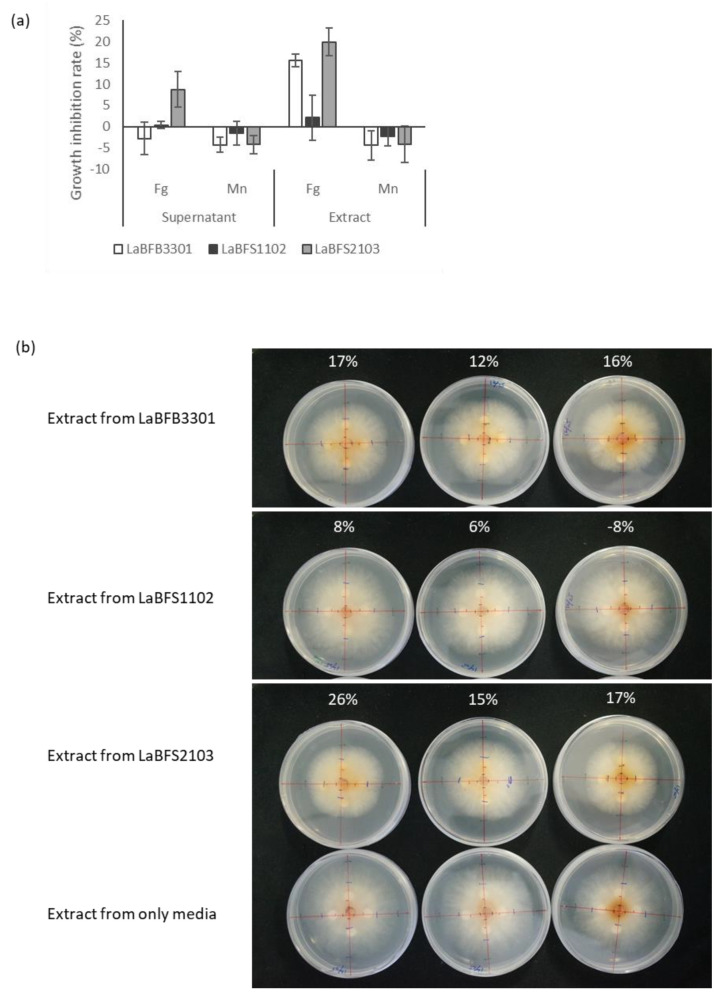
Growth inhibition test of cell-free supernatant and methanolic extracts of three endophytic bacteria, LaBFB3301, LaBFS1102 and LaBFS2103 against mycelial growth of *F. graminearum* (Fg) and *M. nivale* (Mn) after 3-day culture. (**a**) Mean growth inhibition rate with SE as error bars; (**b**) cultured plates of *F. graminearum* on day 3. The above number represents the growth inhibition rate.

Among all the candidates, we selected three isolates, LaBFB3301 (N°2 *Luteibacter rhizovicinus)*, LaBFS1102 (N°3 *Paraburkholderia fungorum)* and LaBFS2103 (N°16 *Comamonas denitrificans)*, as our model strains. They rapidly grew in nutrient broth liquid culture, and their extracts reacted with Dragendorff’s reagent (Figure S2). The results obtained in diffusion assays of the cell-free supernatant of the culture broth or the methanolic extracts showed that the methanolic extracts from LaBFB3301 N°2 *Luteibacter rhizovicinus* and LaFS2103 N°16 *Comamonas denitrificans* delayed the growth of *F. graminearum* colonies on potato dextrose agar (PDA), with mean inhibition rates of 15.5% and 19.9%, respectively (Figure 10a,b). All the samples from LaBFS1102 N°3 *Paraburkholderia fungorum* had no or slight effects on the growth of the fungus (Figure 10a).

### 3.6. Untargeted LC-MS/MS Analysis

The extracts were analyzed for the identification of molecules using untargeted LC-MS/MS analysis. Liquid chromatography coupled with high-resolution mass spectrometry (LC-MS) analysis was performed on methanolic extracts of the three endophytic bacteria strains displaying signals on HPTLC for Dragendorff’s reagent (LaBFS1102, LaBFB3301, LaBFS2103) using the same analytical conditions as previously published [26]. LC-MS analysis performed in positive ion mode confirmed the absence of lycorine and galantamine in the methanolic extracts, as previously observed with the HPTLC analysis (Figure S3).

**Figure 11:**
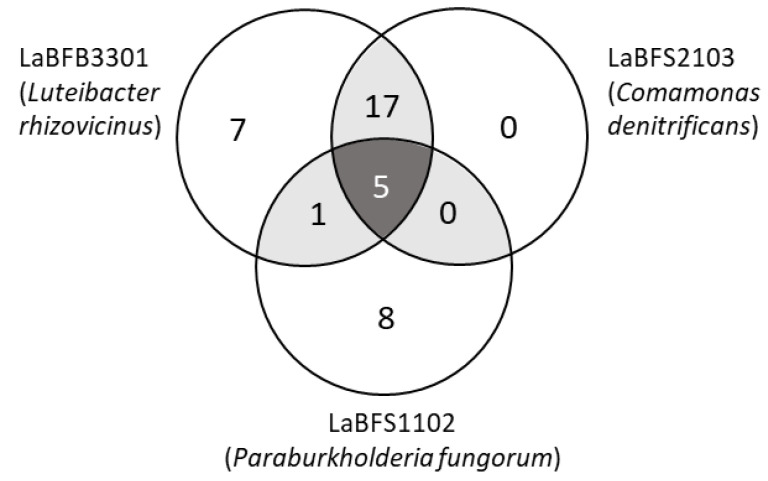
Venn diagram of shared and unique peaks of metabolites enriched and identified in the methanolic extract of the three strains LaBFB 3301, LaBFS1102 and LaBFS2103, using HPLC-Q-Exactive-Orbitrap^®^.

LC-MS profiling generated an extensive mass list corresponding to a wide variety of metabolites in the endophytic extracts from *L. aestivum* ‘Gravety giant’ bulb (LaBFB3301) and from *L. aestivum* ‘Gravety giant’ shoot (LaBFS1102, LaBFS2103), using HPLC-Q-Exactive-Orbitrap^®^. Using the Compound Discoverer software led to the identification of 38 peaks enriched in the methanolic extracts of the bacterial pellets (Figure 11, Table 2), thanks to MS/MS spectra comparison with online databases such as Mzcloud.

Seven, eight and zero compounds were specific to LaBFS1102, LaBFB3301 and LaBFS2103, respectively. Additionally, 17 compounds were shared commonly between LaBFB3301 and LaBFS2103, and only one compound (6,7-dihydro-5H-dibenzo diazepin-6-one) was shared between LaBFB3301 and LaBFS1102. Five molecules (adenosine 5′-monophosphate, palmitoyl ethanolamide, oleoyl ethanolamide, nicotinamide adenine dinucleotide, azobenzene) were identified in the three endophytic extracts.

**Figure 12:**
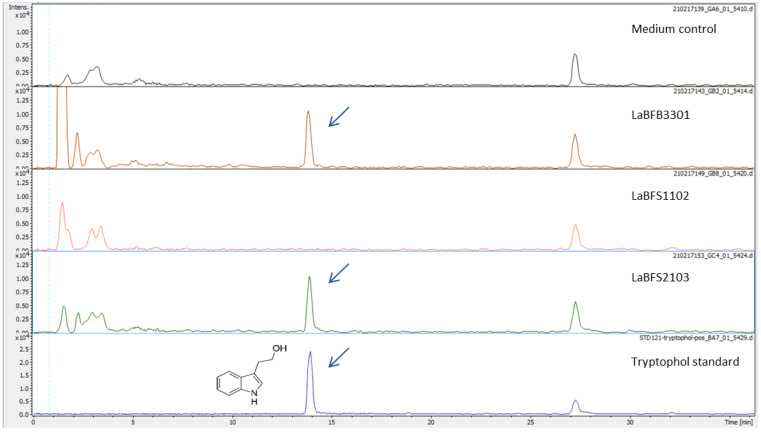
Ion chromatogram of *m/z* 162.0913 ± 0.02 for tryptophol found in the methanolic extracts of LaBFB3301 and LaBFS2103.

In the 17 shared compounds between LaBFB3301 and LaBFS2103, we identified the indolic alkaloid tryptophol or l,3-(2-hydroxyethyl) indole, both by MS analysis (C_10_H_11_NO, [M + H]^+^ 162.0913) and by retention time (13.9 min) (Figure 12).

This result was confirmed using the commercial standard tryptophol. The same peaks and fragments were highlighted in all the triplicates of LaBFB3301 and LaBFS2103 samples (Figure S4). Tryptophol is a precursor of various indole alkaloids in plants and microorganisms [29]. The origin of tryptophol is closely related to the tryptophan metabolic pathway. However, the amino acid tryptophan (C_11_H_12_N_2_O_2_, [M + H]^+^ 205.0972) was not identified in the three endophytic bacteria strains. The peak of tryptophol was observed at 13.8–14.0 min with *m/z* 162.0913 [M + H]^+^, which had MS2 fragments with *m/z* 143, 130 and 115. Moreover, indole-3-acetic acid (IAA), which shares precursors of tryptophol and takes part in plant growth promotion effect, was not found in any samples, even compared with the standard compound.

## 4. Discussion

This research was conducted in order to investigate the diversity of endophytic culturable bacteria collected from three organs of *L. aestivum* ‘Gravity giant’ that were not cultured in sterile conditions. Different strains of endophytic bacteria were selected for the evaluation of their capabilities for producing alkaloids and displaying antagonistic effects against wheat fungal pathogens.

Endophytic bacteria and fungi have been isolated from many plant species, but only a few Amaryllidaceae plants have been studied thus far. The plants that have been investigated are *Narcissus tazetta* [25], *Crinum macowanii* [30,31,32] and *Lycoris radiata* [33]. The latest example was reported by Spina et al. [26], with in vitro bulblets of *L. aestivum*. In our study, the bulbs harbor a low number of cultivable bacterial strains, which is consistent with the observation of the few endophytic bacteria isolated from in vitro bulblets of *L. aestivum* [26]. This result could be associated with the accumulation of alkaloids such as galantamine, which is highest in the bulbs of *Leucojum* plants [34]. Moreover, it is known that in plant multiplication organs, bacterial loads are generally lower than in other parts of the plant. In contrast, the roots were found to contain the highest number of bacterial endophytes, probably explained by the contact of the roots with a large reservoir of bacteria in the soil [6,8,35].

The data collected in our study show that the dominant bacterial phyla in *L. aestivum* ‘Gravety giant’ among culturable endophytes are Proteobacteria (51.4%), Actinobacteria (23.9%), Firmicutes (22.5%) and Bacteroidetes (2.2%). A similar distribution was reported for *Lycoris radiata* [33] or *Zea mays* [36]. When focusing on the taxonomical distribution of endophytic bacteria throughout plant tissues, no genus was shared among the three different tissues we analyzed in *Leucojum aestivum*. This seems to be common, at least with *Lycoris radiata*, for which the analysis was performed among five different organs (flower, scape, leaf, bulb and root) [33]. This is an important point for further studies that aim to identify bacterial strains with some specific properties, and a relationship between plant metabolomic profiles and bacterial diversity might be established.

Amaryllidaceae plants seem to share a similar pattern of endophytic bacterial diversity. A survey carried out in seven different studies (including our study) showed that the most frequently isolated genera were *Bacillus* (5/7), followed by *Acinetobacter*, *Novosphingobium* and *Pseudomonas* (3/7) (Table S2). When going deeper in detail and when focusing on different plant tissues, we could highlight similarities but also some interesting differences. The leaf endophytic bacteria, *Arthrobacter*, *Luteibacter* and *Pseudomonas*, were common to other Amaryllidaceae plant leaf tissues [32,33]. Concerning roots, *Bacillus* and *Paenibacillus* genera were predominant in *Leucojum aestivum* as well as in *Lycoris radiata* [33]. The most interesting point is that *Bacillus*, mostly isolated from bulbs in previous studies, could not be highlighted in our *Leucojum aestivum* ‘Gravety giant’ bulbs. For example, rice seeds harbor mainly *Bacillus* during maturation [37], which was assumed because of the hardness and dormancy of the organ. In other studies, seed endophytes could colonize the next-generation seedlings as well as their rhizosphere [38]. In this study, we used the bulbs under germination, which can be considered as after the break of dormancy. The developmental stage of bulbs we used could be related to the absence of *Bacillus* endophytes.

It is known that endophytic microorganisms are able to synthesize a variety of specialized metabolites with application in agriculture and pharmaceutical industries [39]. Endophytic bacteria belonging to *Bacillus* and *Streptomyces* genera are well known for their metabolic capability to produce various bioactive compounds as well as their function of plant protection [40]. An endophytic strain of *Paenibacillus amylolyticus* was reported to produce dipicolinic acid with antibacterial activity [41] and *Paraburkholderia fungorum* was reported as an endophyte of rice, and it showed plant growth promotion in strawberry [42,43]. *Chitinophaga ginsengisegetis* was reported as a root endophyte in grapevine [44]. *Rahnella aquatilis* was also isolated from *Crinum macowanii* Baker [31], but it was suggested as a pathogen in onion bulb [45]. *Luteibacter rhizovicinus* was found in apple shoots, and displays plant growth promotion properties in barley [46]. The genus *Variovorax* is known for its metabolic diversity and for its plant growth-promoting function [47]. *Staphylococcus* is a well-known genus since some of its species are human pathogens, but this genus is also isolated as endophytes. Rebotiloe et al. [30] isolated bacterial endophytes from *Crinum macowanii* Baker, and three isolates among the total of five isolates were *Staphylococcus* species. They also observed moderate antibacterial activities from those strains.

In this work, the results showed some isolates displaying antagonism against wheat fungal pathogens. Interestingly, all the isolates belonging to *Paraburkholderia fungorum* that were isolated from the shoot and root tissues showed better and less various mycelial growth inhibition. In the genus Paraburkholderia, there is a beneficial endophytic strain named *P. phytofirmans* PsJN. This strain has the capability to promote plant growth and mitigate biotic/abiotic stress in a wide range of host plants by producing various compounds [48]. However, because the methanolic extracts and filtrate of the culture broth of this strain did not show antagonism for the same pathogens, it might be possible that the amounts of bioactive compounds were too low in those samples or that their production relies on induction by the presence of target microorganisms.

The chemical analysis of the methanolic extract of endophytic bacteria strains of shoot and bulbs showed no detection of Amaryllidaceae alkaloids such as galanthamine or lycorine. However, interestingly, two isolates of the endophytic bacteria, *Luteibacter rhizovicinus* and *Commamonas denitrificans*, contained the indolic alkaloid tryptophol. These two strains displayed an inhibitory effect against the growth of *F. graminearum* colonies, while *Paraburkholderia fungorum*, which did not synthesize tryptophol, showed no antagonism activity against this wheat fungal pathogen. This molecule is a precursor of different indole alkaloids in plants and microorganisms [29]. However, indole-3-acetic acid, which shared precursors of tryptophol and took part in the plant growth promotion effect, was not found in the three samples, even compared with the standard compound. On the other hand, IAA was produced by endophytic *Luteibacter rhizobicinus*, and the strain did not have harmful effects on plant growth [46]. Tryptophol itself has been found in plants and microorganisms and was also discovered as one of the quorum-sensing molecules in fungi. It might be possible that it is involved in the induction of cell apoptosis in specific fungi [49]. Tryptophol also has a growth promotion effect in roots and leaves of plants [29]. It is of interest to confirm if this compound relates to the observed antifungal activity and also to search its derivatives in methanolic extracts. Diketopiperazines having antimicrobial activities were detected in *Comamonas testosteroni*, isolated from nematodes [50].

## 5. Conclusions

This study showed for the first time that bacterial endophytic communities are different in bulbs, roots and shoots of *L. aestivum* ‘Gravety giant’. The indolic alkaloid tryptophol, detected only in the strains of *Luteibacter rhizovivinus* (LaBFB3301) and *Comamonas denitrificans* (LaBFS2103), showing an inhibitory effect against the growth of *F. graminearum* colonies, could be a potential agent to preserve plants against bioaggressors. In addition, it might be interesting to characterize the plant growth promotion effect of LaBFB3301 and LaBFS2103 strains in future research.

## Figures and Tables

**Table 1:** List of strains of which extract (possibly) reacted to Dragendorff’s reagent.

Source Tissues	Strain	GenBank Accession	Blast Top Hit	E Value	Inhibition Rate vs. Fg (Mean +/− SE)	Inhibition Rate vs. Mn (Mean +/− SE)	Dragendorff
Shoot	LaBFS1102	OL307020	*Paraburkholderia fungorum* strain LMG 16225	0	41.91 ± 0.19	56.69 ± 0.12	Yes
	LaBFS1106	OL307021	*Patulibacter ginsengiterrae* strain P4–5	0	-	-	Yes
	LaBFS1107	OL307022	*Chitinophaga ginsengisegetis* strain Gsoil 040	0	26.57 ± 0.67	14.54 ± 2.3	Yes
	LaBFS1112	OL307023	*Variovorax ginsengisoli* strain Gsoil 3165	0	0 ± 0.2	9.15 ± 0.76	Yes
	LaBFS1201	OL307024	*Staphylococcus warneri* strain AW 25	0	37.71 ± 0.57	29.73 ± 3.06	Yes
	LaBFS1208		*Mycolicibacterium setense* strain CIP 109395	1 × 10^−151^	2.81 ± 0.74	8.87 ± 0.82	Yes
	LaBFS1307	OL307027	*Microbacterium luteolum* strain IFO 15074	0	4.79 ± 0.95	4.17 ± 1.21	Yes
	LaBFS2103	OL307031	*Comamonas denitrificans* strain 123	0	43.51 ± 0.09	58.82 ± 1.19	Yes
	LaBFS2201		*Microbacterium arthrosphaerae* strain CC-VM-Y	7 × 10^−55^	−0.8 ± 0.06	6.17 ± 0.16	Yes
	LaBFS3105	OL307062	*Variovorax paradoxus* strain NBRC 15149	0	3.4 ± 0.23	7.54 ± 2.97	Yes
	LaBFS3313	OL307078	*Arthrobacter oryzae* strain KV-651	0	30.73 ± 0.96	18.46 ± 3.11	Not performed
Bulb	LaBFB1102	OL307013	*Paenibacillus amylolyticus* strain JCM 9906	0	−0.4 ± 0.12	0 ± 0	Yes
	LaBFB1103	OL307014	*Streptomyces canescens* strain DSM 40001	0	49.59 ± 0.79	59.85 ± 0.82	Not performed
	LaBFB1301	OL307015	*Patulibacter ginsengiterrae* strain P4–5	0	2.99 ± 1.04	32.61 ± 0.36	Yes
	LaBFB1302	OL307016	*Patulibacter ginsengiterrae* strain P4–5	0	1.6 ± 0.35	32.62 ± 0.91	Yes
	LaBFB3301	OL307019	*Luteibacter rhizovicinus* strain LJ96	0	37.32 ± 0.2	48.06 ± 1.01	Yes

The growth inhibition rates against *F. graminearum* and *M. nivale* are also shown in the right columns (means +/− SE).

**Table 2:** List of metabolites detected in the methanolic extracts of the three strains LaBFB3301, LaBFS1102 and LaBFS2103, using UHPLC-Q-Exactive-Orbitrap, combined with the identification using the software, Compound Discoverer (Thermo).

Enrichment in the Samples (Compared with Medium Control)	Compound Name	Formula	Molecular Weight	Error (ppm)	RT (Min)
LaBFB3301 unique	N2,1-Diphenyl-6-imino-1,6-dihydro-1,3,5-triazine-2,4-diamine	C_15_H_14_N_6_	278.12676		7.16
	O-Arachidonoyl ethanolamine	C_22_H_37_NO_2_	347.28231	0.34	33.43
	L(-)-Carnitine	C_7_H_15_NO_3_	161.10521	0.10	1.64
	Adenine	C_5_H_5_N_5_	135.05452	0.18	1.84
	2′-O-Methyladenosine	C_11_H_15_N_5_O_4_	281.11252	0.41	3.11
	Betulin	C_30_H_50_O_2_	442.38107	0.02	35.83
	R-Palmitoyl-(2-methyl) ethanolamide	C_19_H_39_NO_2_	313.29806		27.45
LaBFS1102 unique	16-Heptadecyne-1,2,4-triol	C_17_H_32_O_3_	284.23524	0.33	32.86
	Ethyl oleate	C_20_H_38_O_2_	310.28724	0.19	38.96
	Palmitelaidic acid methyl ester	C_17_H_32_O_2_	268.2403		34.74
	Guanine	C_5_H_5_N_5_O	151.04933	0.53	2.66
	Ethyl palmitoleate	C_18_H_34_O_2_	282.25602	0.49	35.39
	cis-12-Octadecenoic acid methyl ester	C_19_H_36_O_2_	296.27171	0.61	36.45
	3-amino-2-phenyl-2H-pyrazolo [4,3-c]pyridine-4,6-diol	C_12_H_10_N_4_O_2_	242.0804	0.10	16.44
	1,4:3,6-Dianhydro-2-(benzoylamino)-2,5-dideoxy-5-{[4-(3-fluorophenyl)-2-pyrimidinyl]amino}-L-iditol	C_23_H_21_FN_4_O_3_	420.15888		30.35
Shared peaks between LaBFB3301 and LaBFS1203	6,7-dihydro-5H-dibenzo[d,f][1,3]diazepin-6-one	C_13_H_10_N_2_O	210.07936		20.64
Shared peaks between LaBFB3301 and LaBFS2103	L-Histidine	C_6_H_9_N_3_O_2_	155.06942	0.52	1.52
	S-Adenosylhomocysteine	C_14_H_20_N_6_O_5_S	384.12149	0.26	1.85
	Linoleoyl Ethanolamide	C_20_H_37_NO_2_	323.28237	1.14	32.46
	Propionylcarnitine	C_10_H_19_NO_4_	217.1315	0.42	1.97
	Stearoyl ethanolamide	C_20_H_41_NO_2_	327.31365	0.24	34.99
	N6-Acetyl-L-lysine	C_8_H_16_N_2_O_3_	188.11604	0.32	1.80
	3-(2-Hydroxyethyl)indole	C_10_H_11_NO	161.08402	0.50	13.90
	9(Z),11(E),13(E)-Octadecatrienoic Acid methyl ester	C_19_H_32_O_2_	292.24043	0.68	35.25
	Cytidine 5′-diphosphocholine	C_14_H_26_N_4_O_11_P_2_	488.10714	0.33	1.83
	Hypoxanthine	C_5_H_4_N_4_O	136.03845	0.45	2.10
	L-Glutathione oxidized	C_20_H_32_N_6_O_12_S_2_	612.15152	0.72	195
	Imidazolelactic acid	C_6_H_8_N_2_O_3_	156.05344	0.33	1.81
	UDP-N-acetylglucosamine	C_17_H_27_N_3_O_17_P_2_	607.08158	0.31	2.40
	11(Z),14(Z),17(Z)-Eicosatrienoic acid	C_20_H_34_O_2_	306.25594	0.19	35.05
	Υ-Glutamylcysteine	C_8_H_14_N_2_O_5_S	250.06235	0.03	2.01
	Biotin	C_10_H_16_N_2_O_3_S	244.0882	0.15	10.72
	Ergosterol peroxide	C_28_H_44_O_3_	428.32901	0.08	34.95
Shared peaks among the three strains	Adenosine 5′-monophosphate	C_10_H_14_N_5_O_7_P	347.06301	0.22	1.88
	Palmitoyl ethanolamide	C_18_H_37_NO_2_	299.28252	0.30	33.11
	Oleoyl ethanolamide	C_20_H_39_NO_2_	325.29805	0.05	33.69
	Nicotinamide adenine dinucleotide (NAD+)	C_21_H_27_N_7_O_14_P_2_	663.10893	0.29	1.86
	Azobenzene	C_12_H_10_N_2_	182.08434	0.32	10.46

## Supplementary Materials

The following supporting information can be downloaded at: https://www.mdpi.com/article/10.3390/microorganisms10102089/s1, Table S1: Bacterial isolates of *Leucojum aestivum* ‘Gravety giant’ endophytes. Table S2: Bacterial genera found as endophytes in the studies using Amaryllidaceae plants. Figure S1: (**a**) Sampled plants after washing with running tap water; (**b**) a segment of the bulb of plant 2. Figure S2: The plates of HPTLC of the 69 endophyte extracts after Dragendorff’s reagent spraying. Figure S3: Ion chromatograms of *m/z* 288.1266 ± 0.01 for lycorine and *m/z* 288.1564 ± 0.01 for galanthamine. Figure S4: Identification of tryptophol in the extract of LaBFB3301 and LaBFS2103.
