# Supplementary material for: Screening of Endophytic Bacteria of Leucojum aestivum ‘Gravety Giant’ as a Potential Source of Alkaloids and as Antagonist to Some Plant Fungal Pathogens
Source: Microorganisms. 2022 Oct 21;10(10):2089. doi: 10.3390/microorganisms10102089 (PMC9609000; doi:10.3390/microorganisms10102089)
Supplement: Supplementary file 1 [file microorganisms-10-02089-s001.zip › microorganisms-1958442-supplementary.pdf]

Screening of endophytic bacteria of *Leucojum aestivum* 'Gravety giant' as a potential source of alkaloids and as antagonist to some plant fungal pathogens

Yuka Munakata[1,2], Rosella Spina[1,2], Sophie Slezack-Deschaumes[1], Julie Genestier[1], Alain Hehn[1], Dominique Laurain-Mattar[1,2,*]

[1] Université de Lorraine – INRAE, LAE, F-54000 Nancy, France;

[2] Université de Lorraine – CNRS, L2CM, F-54000 Nancy, France;

* Correspondence: dominique.mattar@univ-lorraine.fr (D.L.M)

Table S1. Bacterial isolates of *Leucojum aestivum* 'Gravety giant' endophytes.

				Blast search result					
**Plant**	**Tissue**	**Strain**	**Genbank Accession**	**Blast top hit**	**E Value**	**Tax Id**	**Genus**	**Order**	**Phylum**
1	Bulb	LaBFB1102	OL307013	Paenibacillus amylolyticus strain JCM 9906	0	1451	Paenibacillus	Bacillales	Firmicutes
1	Bulb	LaBFB1103	OL307014	Streptomyces canescens strain DSM 40001	0	53445	Streptomyces	Streptomycetales	Actinobacteria
1	Bulb	LaBFB1301	OL307015	Patulibacter ginsengiterrae strain P4-5	0	556890	Patulibacter	Solirubrobacterales	Actinobacteria
1	Bulb	LaBFB1302	OL307016	Patulibacter ginsengiterrae strain P4-5	0	556890	Patulibacter	Solirubrobacterales	Actinobacteria
1	Bulb	LaBFB1601	OL307017	Mycobacterium hackensackense strain 147-0552	0	228909	Mycobacterium	Corynebacteriales	Actinobacteria
1	Root	LaBFR1102a	OL305761	Bacillus mycoides strain 273	0	1405	Bacillus	Bacillales	Firmicutes
1	Root	LaBFR1103	OL305762	Paraburkholderia fungorum strain LMG 16225	0	134537	Paraburkholderia	Burkholderiales	Proteobacteria
1	Root	LaBFR1104	OL305763	Paenibacillus populi strain LAM0705	0	1473572	Paenibacillus	Bacillales	Firmicutes
1	Root	LaBFR1105	OL305764	[Brevibacterium] frigoritolerans strain DSM 8801	0	450367	Bacillus	Bacillales	Firmicutes
1	Root	LaBFR1107	OL305765	Streptomyces canescens strain DSM 40001	0	53445	Streptomyces	Streptomycetales	Actinobacteria
1	Root	LaBFR1108c	OL305766	Bacillus simplex NBRC 15720 = DSM 1321	0	1349754	Peribacillus	Bacillales	Firmicutes
1	Root	LaBFR1109	OL305767	Paraburkholderia fungorum strain LMG 16225	0	134537	Paraburkholderia	Burkholderiales	Proteobacteria
1	Root	LaBFR1113a	OL305768	Bacillus simplex NBRC 15720 = DSM 1321	0	1349754	Peribacillus	Bacillales	Firmicutes
1	Root	LaBFR1113b	OL305769	Bacillus idriensis strain SMC 4352-2	0	324768	Bacillus	Bacillales	Firmicutes
1	Root	LaBFR1114	OL305770	Bacillus idriensis strain SMC 4352-2	0	324768	Bacillus	Bacillales	Firmicutes
1	Root	LaBFR1201	OL305771	Bacillus idriensis strain SMC 4352-2	0	324768	Bacillus	Bacillales	Firmicutes
1	Root	LaBFR1204a	-	Bacillus licheniformis strain DSM 13	9,00E-69	1402	Bacillus	Bacillales	Firmicutes
1	Root	LaBFR1204b	OL305772	Moraxella osloensis strain DSM 6998	0	34062	Moraxella	Pseudomonadales	Proteobacteria
1	Root	LaBFR1205a	OL305773	Nocardioides cavernae strain YIM A1136	0	1921566	Nocardioides	Propionibacteriales	Actinobacteria
1	Root	LaBFR1205b	-	Bacillus licheniformis strain DSM 13	3,00E-69	1402	Bacillus	Bacillales	Firmicutes
1	Root	LaBFR1206	OL305774	Streptomyces bottropensis ATCC 25435	0	1054862	Streptomyces	Streptomycetales	Actinobacteria
1	Root	LaBFR1207	OL305775	Microbacterium natoriense strain TNJL143-2	0	284570	Microbacterium	Micrococcales	Actinobacteria
1	Root	LaBFR1301b	OL305776	Rathayibacter festucae strain VKM Ac-1390	0	110937	Rathayibacter	Micrococcales	Actinobacteria
1	Root	LaBFR1303a	-	[Brevibacterium] frigoritolerans strain DSM 8801	0	450367	Bacillus	Bacillales	Firmicutes
1	Root	LaBFR1303b	OL305777	[Brevibacterium] frigoritolerans strain DSM 8801	0	450367	Bacillus	Bacillales	Firmicutes
1	Root	LaBFR1304b	OL305778	Paenibacillus endophyticus strain PECAE04	0	1294268	Paenibacillus	Bacillales	Firmicutes
1	Root	LaBFR1305a	OL305779	Staphylococcus warneri strain AW 25	0	1292	Staphylococcus	Bacillales	Firmicutes
1	Root	LaBFR1305b	OL305780	Paenibacillus endophyticus strain PECAE04	0	1294268	Paenibacillus	Bacillales	Firmicutes
1	Root	LaBFR1307a	OL305781	Caulobacter rhizosphaerae strain 7F14	0	2010972	Caulobacter	Caulobacterales	Proteobacteria
1	Root	LaBFR1308	OL305782	Labrys methylaminiphilus JLW10	0	1096091	Labrys	Hyphomicrobiales	Proteobacteria
1	Root	LaBFR1309	OL305783	Paraburkholderia fungorum strain LMG 16225	0	134537	Paraburkholderia	Burkholderiales	Proteobacteria
1	Root	LaBFR1311	OL305784	Agrobacterium rhizogenes strain ATCC 11325	0	359	Agrobacterium	Hyphomicrobiales	Proteobacteria
1	Shoot	LaBFS1102	OL307020	Paraburkholderia fungorum strain LMG 16225	0	134537	Paraburkholderia	Burkholderiales	Proteobacteria
1	Shoot	LaBFS1106	OL307021	Patulibacter ginsengiterrae strain P4-5	0	556890	Patulibacter	Solirubrobacterales	Actinobacteria
1	Shoot	LaBFS1107	OL307022	Chitinophaga ginsengisegetis strain Gsoil 040	0	393003	Chitinophaga	Chitinophagales	Bacteroidetes
1	Shoot	LaBFS1112	OL307023	Variovorax ginsengisoli strain Gsoil 3165	0	363844	Variovorax	Burkholderiales	Proteobacteria
1	Shoot	LaBFS1201	OL307024	Staphylococcus warneri strain AW 25	0	1292	Staphylococcus	Bacillales	Firmicutes
1	Shoot	LaBFS1206	OL307025	Achromobacter marplatensis strain B2	0	470868	Achromobacter	Burkholderiales	Proteobacteria
1	Shoot	LaBFS1208	-	Mycolicibacterium setense strain CIP 109395	1,00E-151	431269	Mycolicibacterium	Corynebacteriales	Actinobacteria
1	Shoot	LaBFS1303	OL307026	Luteibacter jiangsuensis strain JW-64-1	0	637577	Luteibacter	Xanthomonadales	Proteobacteria
1	Shoot	LaBFS1306	-	Dyella terrae strain JS14-6	0	522259	Dyella	Xanthomonadales	Proteobacteria
1	Shoot	LaBFS1307	OL307027	Microbacterium luteolum strain IFO 15074	0	69367	Microbacterium	Micrococcales	Actinobacteria
1	Shoot	LaBFS1311	OL307028	Roseateles depolymerans strain 61A	9,00E-157	76731	Roseateles	Burkholderiales	Proteobacteria

2	Bulb	LaBFB2301	OL307018	Agrobacterium rhizogenes strain ATCC 11325	0	359	Agrobacterium	Hyphomicrobiales	Proteobacteria
2	Root	LaBFR2101	OL305785	Microbacterium maritypicum strain DSM 12512	0	300020	Microbacterium	Micrococcales	Actinobacteria
2	Root	LaBFR2103	OL305786	Streptomyces phaeochromogenes strain NBRC 12898	0	1923	Streptomyces	Streptomycetales	Actinobacteria
2	Root	LaBFR2105a	OL305787	Phyllobacterium ifriqiyense strain STM 370	0	314238	Phyllobacterium	Hyphomicrobiales	Proteobacteria
2	Root	LaBFR2105b	OL305788	[Brevibacterium] frigoritolerans strain DSM 8801	0	450367	Bacillus	Bacillales	Firmicutes
2	Root	LaBFR2105c	OL305789	Bacillus simplex NBRC 15720 = DSM 1321	0	1349754	Peribacillus	Bacillales	Firmicutes
2	Root	LaBFR2107	OL305790	Paenibacillus harenae strain B519	0	306543	Paenibacillus	Bacillales	Firmicutes
2	Root	LaBFR2108	OL305791	Microlunatus nigridraconis strain CPCC 203993	0	1710544	Microlunatus	Propionibacteriales	Actinobacteria
2	Root	LaBFR2201	OL305792	Leifsonia naganoensis strain DB103	0	150025	Leifsonia	Micrococcales	Actinobacteria
2	Root	LaBFR2202	OL305793	Mycobacterium hackensackense strain 147-0552	0	228909	Mycobacterium	Corynebacteriales	Actinobacteria
2	Root	LaBFR2204a	OL305794	Bacillus subtilis strain IAM 12118	0	1423	Bacillus	Bacillales	Firmicutes
2	Root	LaBFR2301	OL305795	Microbacterium natoriense strain TNJL143-2	0	284570	Microbacterium	Micrococcales	Actinobacteria
2	Root	LaBFR2303	-	Pedobacter ginsengisoli strain Gsoil 104	0	363852	Pedobacter	Sphingobacteriales	Bacteroidetes
2	Shoot	LaBFS2101	OL307029	Rahnella aquatilis CIP 78.65 = ATCC 33071 strain DSM	0	745277	Rahnella	Enterobacterales	Proteobacteria
2	Shoot	LaBFS2102	OL307030	Rahnella aquatilis CIP 78.65 = ATCC 33071 strain DSM	0	745277	Rahnella	Enterobacterales	Proteobacteria
2	Shoot	LaBFS2103	OL307031	Comamonas denitrificans strain 123	0	117506	Comamonas	Burkholderiales	Proteobacteria
2	Shoot	LaBFS2104	OL307032	Rahnella aquatilis CIP 78.65 = ATCC 33071 strain DSM	0	745277	Rahnella	Enterobacterales	Proteobacteria
2	Shoot	LaBFS2105	OL307033	Rahnella aquatilis CIP 78.65 = ATCC 33071 strain DSM	0	745277	Rahnella	Enterobacterales	Proteobacteria
2	Shoot	LaBFS2109	OL307034	Rahnella aquatilis CIP 78.65 = ATCC 33071 strain DSM	0	745277	Rahnella	Enterobacterales	Proteobacteria
2	Shoot	LaBFS2110	OL307035	Rahnella aquatilis CIP 78.65 = ATCC 33071 strain DSM	0	745277	Rahnella	Enterobacterales	Proteobacteria
2	Shoot	LaBFS2111	OL307036	Rahnella aquatilis CIP 78.65 = ATCC 33071 strain DSM	0	745277	Rahnella	Enterobacterales	Proteobacteria
2	Shoot	LaBFS2112	OL307037	Rahnella aquatilis CIP 78.65 = ATCC 33071 strain DSM	0	745277	Rahnella	Enterobacterales	Proteobacteria
2	Shoot	LaBFS2113	OL307038	Rahnella aquatilis CIP 78.65 = ATCC 33071 strain DSM	0	745277	Rahnella	Enterobacterales	Proteobacteria
2	Shoot	LaBFS2201	-	Microbacterium arthrosphaerae strain CC-VM-Y	7,00E-55	792652	Microbacterium	Micrococcales	Actinobacteria
2	Shoot	LaBFS2202	OL307039	Rahnella aquatilis CIP 78.65 = ATCC 33071 strain DSM	0	745277	Rahnella	Enterobacterales	Proteobacteria
2	Shoot	LaBFS2203	OL307040	Rahnella aquatilis CIP 78.65 = ATCC 33071 strain DSM	0	745277	Rahnella	Enterobacterales	Proteobacteria
2	Shoot	LaBFS2204	OL307041	Rahnella aquatilis CIP 78.65 = ATCC 33071 strain DSM	0	745277	Rahnella	Enterobacterales	Proteobacteria
2	Shoot	LaBFS2205	OL307042	Erwinia persicina strain NBRC 102418	0	55211	Erwinia	Enterobacterales	Proteobacteria
2	Shoot	LaBFS2208	OL307043	Rahnella aquatilis CIP 78.65 = ATCC 33071 strain DSM	0	745277	Rahnella	Enterobacterales	Proteobacteria
2	Shoot	LaBFS2301	OL307044	Rahnella aquatilis CIP 78.65 = ATCC 33071 strain DSM	0	745277	Rahnella	Enterobacterales	Proteobacteria
2	Shoot	LaBFS2302	OL307045	Rahnella aquatilis CIP 78.65 = ATCC 33071 strain DSM	0	745277	Rahnella	Enterobacterales	Proteobacteria
2	Shoot	LaBFS2303	OL307046	Rahnella aquatilis CIP 78.65 = ATCC 33071 strain DSM	0	745277	Rahnella	Enterobacterales	Proteobacteria
2	Shoot	LaBFS2304	OL307047	Rahnella aquatilis CIP 78.65 = ATCC 33071 strain DSM	0	745277	Rahnella	Enterobacterales	Proteobacteria
2	Shoot	LaBFS2305	OL307048	Rahnella aquatilis CIP 78.65 = ATCC 33071 strain DSM	0	745277	Rahnella	Enterobacterales	Proteobacteria
2	Shoot	LaBFS2306	OL307049	Rahnella aquatilis CIP 78.65 = ATCC 33071 strain DSM	0	745277	Rahnella	Enterobacterales	Proteobacteria
2	Shoot	LaBFS2308	OL307050	Rahnella aquatilis CIP 78.65 = ATCC 33071 strain DSM	0	745277	Rahnella	Enterobacterales	Proteobacteria
2	Shoot	LaBFS2309	OL307051	Rahnella aquatilis CIP 78.65 = ATCC 33071 strain DSM	8,00E-127	745277	Rahnella	Enterobacterales	Proteobacteria
2	Shoot	LaBFS2310	OL307052	Rahnella aquatilis CIP 78.65 = ATCC 33071 strain DSM	0	745277	Rahnella	Enterobacterales	Proteobacteria
2	Shoot	LaBFS2313	OL307053	Rahnella aquatilis CIP 78.65 = ATCC 33071 strain DSM	0	745277	Rahnella	Enterobacterales	Proteobacteria
2	Shoot	LaBFS2314	OL307054	Rahnella aquatilis CIP 78.65 = ATCC 33071 strain DSM	0	745277	Rahnella	Enterobacterales	Proteobacteria
2	Shoot	LaBFS2315	OL307055	Rahnella aquatilis CIP 78.65 = ATCC 33071 strain DSM	0	745277	Rahnella	Enterobacterales	Proteobacteria
2	Shoot	LaBFS2316	OL307056	Rahnella aquatilis CIP 78.65 = ATCC 33071 strain DSM	0	745277	Rahnella	Enterobacterales	Proteobacteria
2	Shoot	LaBFS2317	OL307057	Achromobacter marplatensis strain R-46660	0	470868	Achromobacter	Burkholderiales	Proteobacteria
2	Shoot	LaBFS2318	OL307058	Rahnella aquatilis CIP 78.65 = ATCC 33071 strain DSM	0	745277	Rahnella	Enterobacterales	Proteobacteria

3	Bulb	LaBFB3301	OL307019	Luteibacter rhizovicinus strain LJ96	0	242606	Luteibacter	Xanthomonadales	Proteobacteria
3	Root	LaBFR3101a	OL305796	Paenibacillus lautus strain AB236d	0	1401	Paenibacillus	Bacillales	Firmicutes
3	Root	LaBFR3101b	OL305797	Paenarthrobacter nitroguajacolicus strain G2-1	0	211146	Paenarthrobacter	Micrococcales	Actinobacteria
3	Root	LaBFR3102b	OL305798	Paenibacillus lautus strain AB236d	0	1401	Paenibacillus	Bacillales	Firmicutes
3	Root	LaBFR3103	OL305799	Streptomyces canescens strain DSM 40001	0	53445	Streptomyces	Streptomycetales	Actinobacteria
3	Root	LaBFR3104	OL305800	Paenibacillus lautus strain AB236d	0	1401	Paenibacillus	Bacillales	Firmicutes
3	Root	LaBFR3105b	OL305801	Paenibacillus lautus strain AB236d	0	1401	Paenibacillus	Bacillales	Firmicutes
3	Root	LaBFR3106	OL305802	Microbacterium maritypicum strain DSM 12512	0	300020	Microbacterium	Micrococcales	Actinobacteria
3	Root	LaBFR3107a	OL305803	Promicromonospora alba strain 1C-HV12	0	1616110	Promicromonospora	Micrococcales	Actinobacteria
3	Root	LaBFR3107b	OL305804	Promicromonospora alba strain 1C-HV12	0	1616110	Promicromonospora	Micrococcales	Actinobacteria
3	Root	LaBFR3108	OL305805	Paenibacillus lautus strain AB236d	0	1401	Paenibacillus	Bacillales	Firmicutes
3	Root	LaBFR3109	OL305806	Paenibacillus lautus strain AB236d	0	1401	Paenibacillus	Bacillales	Firmicutes
3	Root	LaBFR3110	OL305807	Paenibacillus lautus strain AB236d	0	1401	Paenibacillus	Bacillales	Firmicutes
3	Root	LaBFR3202	OL305808	Bacillus marisflavi strain TF-11	0	189381	Rossellomorea	Bacillales	Firmicutes
3	Root	LaBFR3203	OL305809	Agrobacterium tumefaciens strain IAM 12048	0	358	Agrobacterium	Hyphomicrobiales	Proteobacteria
3	Root	LaBFR3207	OL305810	Inquilinus limosus strain AU476	0	171674	Inquilinus	Rhodospirillales	Proteobacteria
3	Root	LaBFR3209	OL305811	Caulobacter segnis strain MBIC 2835	0	88688	Caulobacter	Caulobacterales	Proteobacteria
3	Root	LaBFR3210	OL305812	Phyllobacterium trifolii strain PETP02	0	300193	Phyllobacterium	Hyphomicrobiales	Proteobacteria
3	Root	LaBFR3211	OL305813	Nocardia rhamnosiphila NBRC 108938 strain 202GMO	0	1223547	Nocardia	Corynebacteriales	Actinobacteria
3	Root	LaBFR3212	OL305814	Staphylococcus epidermidis strain Fussel	0	1282	Staphylococcus	Bacillales	Firmicutes
3	Root	LaBFR3301b	OL305815	Sphingopyxis chilensis strain S37	0	180400	Sphingopyxis	Sphingomonadales	Proteobacteria
3	Root	LaBFR3303	OL305816	Sphingopyxis chilensis strain S37	0	180400	Sphingopyxis	Sphingomonadales	Proteobacteria
3	Root	LaBFR3305a	OL305817	Ensifer adhaerens strain LMG 20216	0	106592	Ensifer	Hyphomicrobiales	Proteobacteria
3	Root	LaBFR3305b	-	Streptomyces canescens strain DSM 40001	2,00E-147	53445	Streptomyces	Streptomycetales	Actinobacteria
3	Root	LaBFR3307	OL305818	Bacillus cereus strain IAM 12605	0	1396	Bacillus	Bacillales	Firmicutes
3	Root	LaBFR3308a	OL305819	Streptomyces canescens strain DSM 40001	0	53445	Streptomyces	Streptomycetales	Actinobacteria
3	Root	LaBFR3308b	OL305820	Streptomyces canescens strain DSM 40001	0	53445	Streptomyces	Streptomycetales	Actinobacteria
3	Root	LaBFR3311	OL305821	Nocardia jinanensis strain 04-5195	0	382504	Nocardia	Corynebacteriales	Actinobacteria
3	Root	LaBFR3312	OL305822	Ensifer adhaerens strain LMG 20216	0	106592	Ensifer	Hyphomicrobiales	Proteobacteria
3	Shoot	LaBFS3102	OL307059	Variovorax guangxiensis strain GXGD002	0	1775474	Variovorax	Burkholderiales	Proteobacteria
3	Shoot	LaBFS3103	OL307060	Luteibacter jiangsuensis strain JW-64-1	0	637577	Luteibacter	Xanthomonadales	Proteobacteria
3	Shoot	LaBFS3104	OL307061	Variovorax paradoxus NBRC 15149	0	1321610	Variovorax	Burkholderiales	Proteobacteria
3	Shoot	LaBFS3105	OL307062	Variovorax paradoxus NBRC 15149	0	1321610	Variovorax	Burkholderiales	Proteobacteria
3	Shoot	LaBFS3108	OL307063	Herbiconiux ginsengi strain wged11	0	381665	Herbiconiux	Micrococcales	Actinobacteria
3	Shoot	LaBFS3109	OL307064	Paraburkholderia fungorum strain LMG 16225	0	134537	Paraburkholderia	Burkholderiales	Proteobacteria
3	Shoot	LaBFS3201	OL307065	Variovorax boronicumulans NBRC 103145	0	1321609	Variovorax	Burkholderiales	Proteobacteria
3	Shoot	LaBFS3203	OL307066	Variovorax boronicumulans NBRC 103145	0	1321609	Variovorax	Burkholderiales	Proteobacteria
3	Shoot	LaBFS3205	OL307067	Pseudomonas brassicacearum strain DBK11	0	930166	Pseudomonas	Pseudomonadales	Proteobacteria
3	Shoot	LaBFS3206	OL307068	Variovorax boronicumulans NBRC 103145	0	1321609	Variovorax	Burkholderiales	Proteobacteria
3	Shoot	LaBFS3208	OL307069	Novosphingobium lindaniclasticum LE124	0	1096930	Novosphingobium	Sphingomonadales	Proteobacteria
3	Shoot	LaBFS3209	OL307070	Variovorax boronicumulans NBRC 103145	0	1321609	Variovorax	Burkholderiales	Proteobacteria
3	Shoot	LaBFS3301	OL307071	Herbaspirillum hiltneri N3	0	1262470	Herbaspirillum	Burkholderiales	Proteobacteria
3	Shoot	LaBFS3302	OL307072	Herbaspirillum hiltneri N3	0	1262470	Herbaspirillum	Burkholderiales	Proteobacteria
3	Shoot	LaBFS3303	OL307073	Leifsonia naganoensis strain DB103	0	150025	Leifsonia	Micrococcales	Actinobacteria
3	Shoot	LaBFS3304	OL307074	Chitinophaga ginsengisegetis strain Gsoil 040	0	393003	Chitinophaga	Chitinophagales	Bacteroidetes
3	Shoot	LaBFS3305	OL307075	Variovorax ginsengisoli strain Gsoil 3165	0	363844	Variovorax	Burkholderiales	Proteobacteria
3	Shoot	LaBFS3306	OL307080	Luteibacter jiangsuensis strain JW-64-1	0	637577	Luteibacter	Xanthomonadales	Proteobacteria
3	Shoot	LaBFS3307	OL307076	Luteibacter jiangsuensis strain JW-64-1	0	637577	Luteibacter	Xanthomonadales	Proteobacteria
3	Shoot	LaBFS3309	OL307077	Curtobacterium citreum strain DSM 20528	0	2036	Curtobacterium	Micrococcales	Actinobacteria
3	Shoot	LaBFS3313	OL307078	Arthrobacter oryzae strain KV-651	0	409290	Arthrobacter	Micrococcales	Actinobacteria
3	Shoot	LaBFS3315	OL307079	Luteibacter jiangsuensis strain JW-64-1	0	637577	Luteibacter	Xanthomonadales	Proteobacteria

Table S2. Bacterial genera found as endophytes in the studies using Amaryllidaceae plants

**Bacterial genus**	**bulb**					**in vitro bulblet**	**leaf**			**root**	
	Lycoris radiata (Liu et al., 2020)	*Crinum macowanii* Baker (Rebotiloe et al., 2018)	*Crinum macowanii* Baker (Sebola et al., 2019)	*Leucojum aestivum* (this study)	*Narcissus tazetta* (Wang et al., 2015b)	*Leucojum aestivum* (Spina et al., 2021)	*Lycoris radiata* (Liu et al., 2020)	*Crinum macowanii* Baker (Sebola et al., 2020)	*Leucojum aestivum* (this study)	*Lycoris radiata* (Liu et al., 2020)	*Leucojum aestivum* (this study)
Achromobacter	0	0	0	0	1	0	0	0	1	0	0
Acinetobacter	0	1	1	0	0	0	1	1	0	1	0
Agrobacterium	0	0	0	1	0	0	0	0	0	0	1
Arthrobacter	0	0	0	0	0	0	0	1	1	0	0
Azoarcus	1	0	0	0	0	0	0	0	0	0	0
Bacillus	1	1	1	0	0	1	1	1	0	1	1
Brachybacterium	0	0	0	0	0	0	0	0	0	0	0
Brevibacterium	0	0	0	0	0	0	1	0	0	0	1
Brevundimonas	0	0	0	0	0	0	0	0	0	1	0
Burkholderia	1	0	1	0	0	0	0	0	0	1	0
Caulobacter	0	0	0	0	0	0	0	0	0	0	1
Chitinophaga	0	0	0	0	0	0	0	0	1	0	0
Cohnella	0	0	0	0	0	0	0	0	0	1	0
Comamonas	0	0	0	0	0	0	0	0	1	0	0
Curtobacterium	0	0	0	0	0	0	0	0	1	0	0
Dyella	0	0	0	0	0	0	0	0	1	0	0
Ensifer	0	0	0	0	0	0	0	0	0	0	1
Enterobacter	1	0	0	0	0	0	0	1	0	1	0
Erwinia	0	0	0	0	0	0	0	0	1	0	0
Fictibacillus	0	0	0	0	0	0	0	0	0	1	0
Herbaspirillum	0	0	0	0	0	0	0	0	1	1	0
Herbiconiux	0	0	0	0	0	0	0	0	1	0	0
Inquilinus	0	0	0	0	0	0	0	0	0	0	1
Labrys	0	0	0	0	0	0	0	0	0	0	1
Leifsonia	0	0	0	0	0	0	0	0	1	0	1
Luteibacter	0	0	0	1	0	0	1	0	1	0	0

Lysobacter	0	0	0	0	0	0	0	0	0	1	0
Methylobacterium	0	0	0	0	0	0	1	0	0	0	0
Microbacterium	1	0	0	0	0	0	0	0	1	0	1
Microlunatus	0	0	0	0	0	0	0	0	0	0	1
Moraxella	0	0	0	0	0	0	0	0	0	0	1
Mycobacterium	0	0	0	1	0	0	0	0	0	0	1
Mycolicibacterium	0	0	0	0	0	0	0	0	1	0	0
Nocardia	0	0	0	0	0	0	0	0	0	0	1
Nocardioides	0	0	0	0	0	0	1	0	0	0	1
Novosphingobium	0	0	1	0	0	0	0	0	1	1	0
Paenarthrobacter	0	0	0	0	0	0	0	0	0	0	1
Paenibacillus	0	0	0	1	0	0	0	0	0	1	1
Pantoea	1	0	0	0	0	0	1	0	0	0	0
Paraburkholderia	0	0	0	0	0	0	0	0	1	0	1
Patulibacter	0	0	0	1	0	0	0	0	1	0	0
Pedobacter	0	0	0	0	0	0	0	0	0	0	1
Phyllobacterium	0	0	0	0	0	0	0	0	0	0	1
Promicromonospora	0	0	0	0	0	0	0	0	0	0	1
Pseudacidovorax	0	0	0	0	0	0	1	0	0	0	0
Pseudomonas	0	0	1	0	0	0	1	1	1	1	0
Rahnella	0	0	1	0	0	0	0	0	1	0	0
Raoultella	0	0	1	0	0	0	0	1	0	0	0
Rathayibacter	0	0	0	0	0	0	0	0	0	0	1
Rhizobium	1	0	0	0	0	0	0	0	0	0	0
Roseateles	0	0	0	0	0	0	0	0	1	1	0
Rosenbergiella	0	0	0	0	0	0	0	0	0	0	0
Sphingobacterium	0	0	0	0	0	0	1	0	0	0	0
Sphingomonas	0	0	0	0	0	0	1	0	0	0	0
Sphingopyxis	0	0	0	0	0	0	0	0	0	0	1
Sporosarcina	0	0	0	0	0	0	1	0	0	0	0
Staphylococcus	0	1	0	0	0	0	0	0	1	0	1
Stenotrophomonas	0	0	0	0	0	0	0	0	0	0	0
Streptomyces	0	0	0	1	0	0	0	0	0	0	1
Variovorax	0	0	0	0	0	0	0	0	1	1	0
Total number of genera	7	3	7	6	1	1	12	6	21	14	24

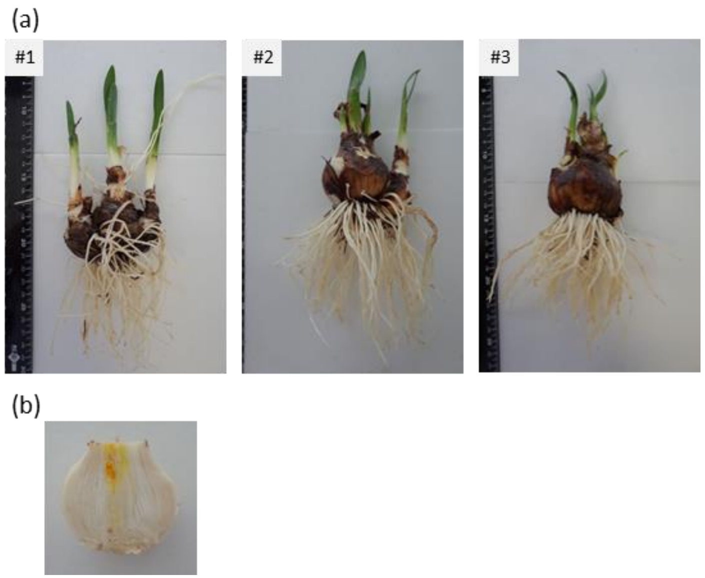

Figure S1. (a) Sampled plants after washing with running tap water; (b) A segment of the bulb of plant 2.

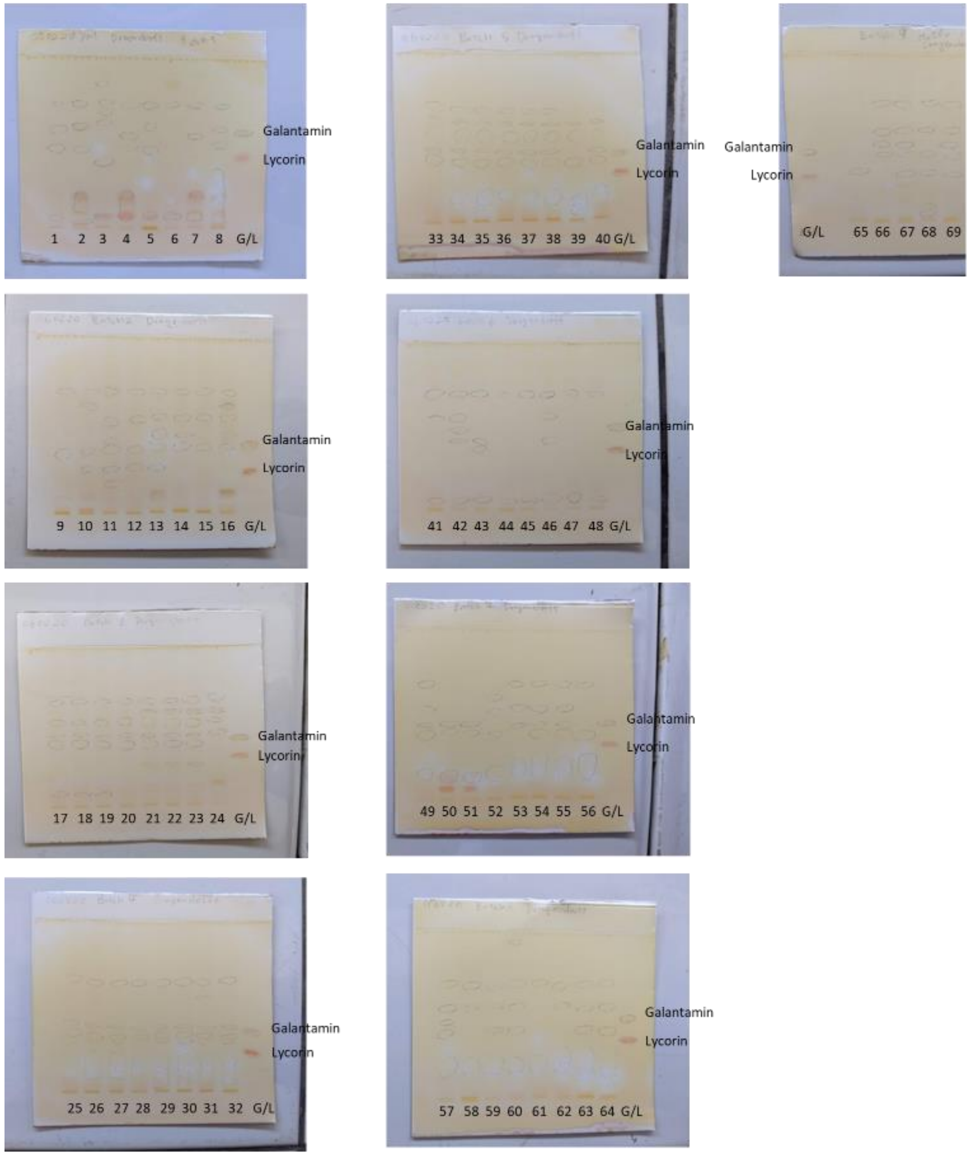

Lane #	TLC batch #	Name	BLAST Top hit
1	1	LaBFB2301	Rhizobium lusitanum strain LrBB45
2	1	LaBFB3301	Luteibacter rhizovicinus strain LJ96
3	1	LaBFS1102	Paraburkholderia fungorum strain LMG 16225
4	1	LaBFS1106	Patulibacter ginsengiterrae strain P4-5
5	1	LaBFS1107	Chitinophaga ginsengisegetis strain Gsoil 040
6	1	LaBFS1112	Variovorax ginsengisoli strain OX0130
7	1	LaBFS1201	Staphylococcus warneri strain af-L2
8	1	LaBFS1203	
9	2	LaBFS1206	Achromobacter marplatensis strain B2
10	2	LaBFS1208	Mycolicibacterium setense strain CIP 109395
11	2	LaBFS1303	Luteibacter jiangsuensis strain JW-64-1
12	2	LaBFS1306	Dyella terrae strain JS14-6
13	2	LaBFS1307	Microbacterium luteolum strain IFO 15074
14	2	LaBFS2101	Rahnella aquatilis strain E53CS3
15	2	LaBFS2102	Rahnella aquatilis strain E53CS3
16	2	LaBFS2103	Comamonas denitrificans strain 123
17	3	LaBFS2104	Rahnella aquatilis strain E53CS3
18	3	LaBFS2105	Rahnella aquatilis strain E53CS3
19	3	LaBFS2109	Rahnella aquatilis strain E53CS3
20	3	LaBFS2110	Rahnella aquatilis strain E53CS3
21	3	LaBFS2111	Rahnella aquatilis strain KM05
22	3	LaBFS2112	Rahnella aquatilis strain E53CS3
23	3	LaBFS2113	Rahnella aquatilis CIP 78.65 = ATCC 33071 strain DSM 4594
24	3	LaBFS2201	Microbacterium arthrosphaerae strain CC-VM-Y
25	4	LaBFS2202	Rahnella aquatilis strain KAAr-197
26	4	LaBFS2203	Rahnella aquatilis CIP 78.65 = ATCC 33071 strain DSM 4594
27	4	LaBFS2204	Rahnella aquatilis strain KM05
28	4	LaBFS2205	Erwinia persicina strain NBRC 102418
29	4	LaBFS2208	Rahnella aquatilis CIP 78.65 = ATCC 33071 strain DSM 4594
30	4	LaBFS2301	Rahnella aquatilis strain D80_CV1R
31	4	LaBFS2302	Rahnella aquatilis strain E53CS3
32	4	LaBFS2303	Rahnella aquatilis strain K9
33	5	LaBFS2304	Rahnella aquatilis strain JM43
34	5	LaBFS2305	Rahnella aquatilis strain JM43
35	5	LaBFS2306	Rahnella aquatilis strain E53CS3
36	5	LaBFS2308	Rahnella aquatilis strain D80_CV1R
37	5	LaBFS2309	Rahnella aquatilis strain K9
38	5	LaBFS2310	Rahnella aquatilis strain JM43
39	5	LaBFS2313	Rahnella aquatilis strain JM43
40	5	LaBFS2314	Rahnella aquatilis strain E53CS3
41	6	LaBFS3102	Variovorax paradoxus NBRC 15149
42	6	LaBFS3103	Luteibacter jiangsuensis strain JW-64-1
43	6	LaBFS3109	Paraburkholderia fungorum strain LMG 16225
44	6	LaBFS3201	Variovorax paradoxus strain 5C-2
45	6	LaBFS3203	Variovorax paradoxus strain 5C-2
46	6	LaBFS3205	Pseudomonas brassicacearum strain DBK11
47	6	LaBFS3206	Variovorax paradoxus strain 5C-2
48	6	LaBFS3301	Herbaspirillum hiltneri strain Field_seedling_213
49	7	LaBFB1102	Paenibacillus amylolyticus strain JCM 9906
50	7	LaBFB1301	Patulibacter ginsengiterrae strain P4-5
51	7	LaBFB1302	Patulibacter ginsengiterrae strain P4-5
52	7	LaBFB1601	Mycobacterium hackensackense strain 147-0552
53	7	LaBFS1311	Roseateles terrae strain CCUG 52222
54	7	LaBFS2315	Rahnella aquatilis strain JM43
55	7	LaBFS2316	Rahnella aquatilis strain E53CS3
56	7	LaBFS2317	Achromobacter marplatensis strain B2
57	8	LaBFS2318	Rahnella aquatilis strain E53CS3
58	8	LaBFS3104	Variovorax paradoxus strain CSUSB
59	8	LaBFS3108	Herbiconiux ginsengi strain wged11
60	8	LaBFS3208	Novosphingobium guangzhouense strain MB49
61	8	LaBFS3209	Variovorax paradoxus strain 5C-2
62	8	LaBFS3302	Herbaspirillum hiltneri strain PT42
63	8	LaBFS3303	Leifsonia naganoensis strain DB103
64	8	LaBFS3304	Chitinophaga ginsengisegetis strain Gsoil 040
65	9	LaBFS3305	Variovorax ginsengisoli strain OX0130
66	9	LaBFS3306	Luteibacter jiangsuensis strain JW-64-1
67	9	LaBFS3307	Luteibacter jiangsuensis strain JW-64-1
68	9	LaBFS3309	Curtobacterium citreum strain DSM 20528
69	9	LaBFS3315	Luteibacter jiangsuensis strain JW-64-1

Figure S2. The plates of HPTLC of the 69 endophyte extracts after Dragendorff's reagent spraying.

The photos were observed after being dried over one night. The lane number in the plate photos corresponds to the first column of the left table. Lycorine and galantamine at 1 mg/mL were applied and developed together with the samples.

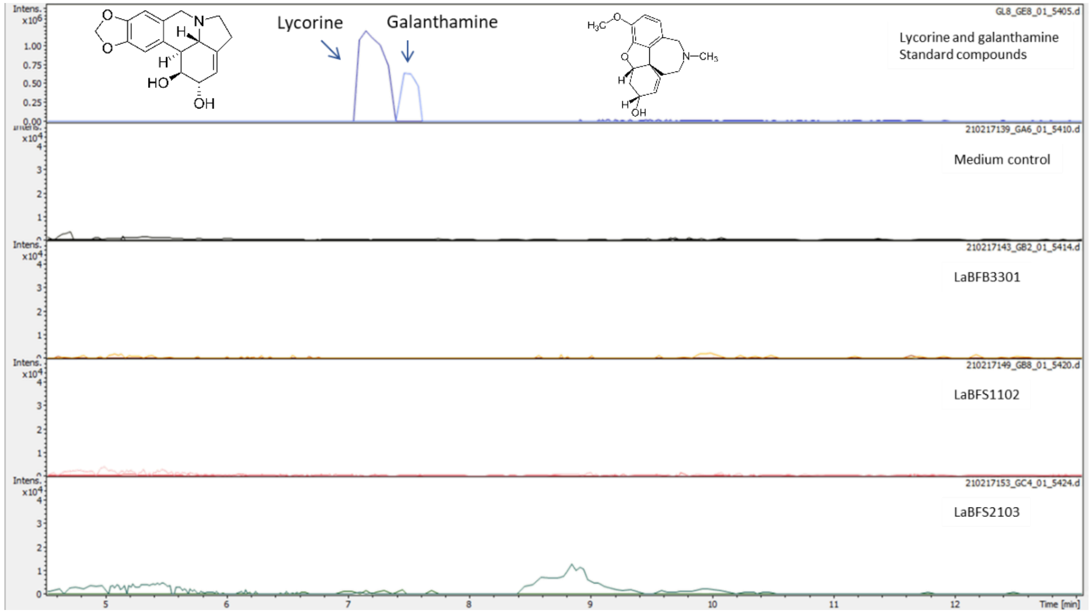

Figure S3. Ion chromatograms of *m/z* 288.1266 ±0.01 for lycorine and *m/z* 288.1564 ±0.01 for galanthamine.

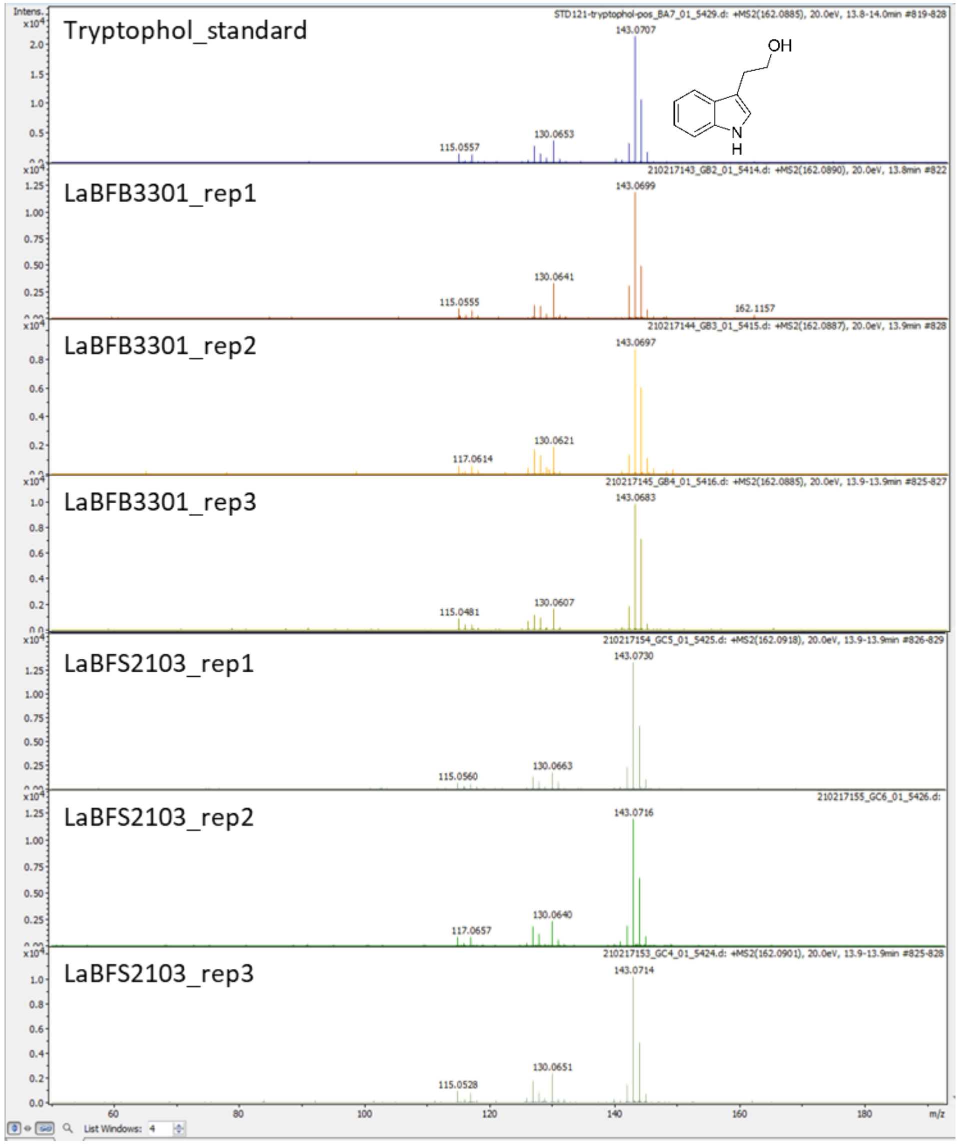

Figure S4. Identification of tryptophol in the extract of LaBFB3301 and LaBFS2103.
